# 3‐Hydroxythiophenol‐Formaldehyde Resin Microspheres Modulated by Sulfhydryl Groups for Highly Efficient Photocatalytic Synthesis of H_2_O_2_

**DOI:** 10.1002/advs.202304948

**Published:** 2023-12-10

**Authors:** Yulu Xu, Xia Hu, Yuyuan Chen, Sijie Lin, Chen Wang, Faliang Gou, Xiaogang Yang, Weiwei Zheng, De‐Kun Ma

**Affiliations:** ^1^ Zhejiang Key Laboratory of Alternative Technologies for Fine Chemicals Process Shaoxing University Shaoxing 312000 China; ^2^ School of Life Science Shaoxing University Shaoxing 312000 China; ^3^ Institute of Materials Science and Devices Suzhou University of Science and Technology Suzhou 215011 China; ^4^ Department of Chemistry Syracuse University Syracuse NY 13244 USA

**Keywords:** 3‐hydroxythiophenol‐formaldehyde resin, H_2_O_2_ production, photocatalytic oxygen reduction reaction, photocatalytic performance, sulfhydryl groups

## Abstract

Resorcinol‐formaldehyde (RF) resin represents a promising visible‐light responding photocatalyst for oxygen reduction reaction (ORR) toward H_2_O_2_ production. However, its photocatalytic ORR activity toward H_2_O_2_ generation is still unsatisfied for practical application. Herein, 3‐hydroxythiophenol‐formaldehyde (3‐HTPF) resin microspheres synthesized through polycondensation reaction between 3‐HTP and formaldehyde at room temperature and subsequent hydrothermal treatment exhibit enhanced photocatalytic ORR activity is reported. The experimental results show that the partial substitution of hydroxy group (─OH) by sulfhydryl one (─SH) through using 3‐HTP to replace resorcinol could slow the rates of nucleation and growth of the resin particles and lead to strongly *π*‐stacked architecture in 3‐HTPF. The introduction of ─SH group can also improve adsorption ability of 3‐HTPF to O_2_ molecules and enhance ORR catalytic activity of the photocatalysts. Stronger built‐in electric field, better adsorption ability to O_2_ molecules, and increased surface catalytic activity collectively boost photocatalytic activity of 3‐HTPF microspheres. As a result, H_2_O_2_ production rate of 2010 µm h^−1^ is achieved over 3‐HTPF microspheres at 273 K, which is 3.4 times larger than that obtained using RF submicrospheres (591 µm h^−1^). The rational substituent group modulation provides a new strategy for designing polymeric photocatalysts at the molecular level toward high‐efficiency artificial photosynthesis.

## Graphical abstract

A “kill three birds with a stone” strategy is reported, namely, the replacement of resorcinol with 3‐hydroxythiophenol can lead to stronger built‐in electric field, better adsorption ability to O_2_ molecules, and increased surface catalytic activity. As a result, H_2_O_2_ production rate for 3‐HTPF microspheres is 3.4 times larger than that obtained on RF submicrospheres.

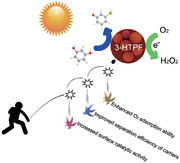

## 1 Introduction

As one of the 100 most important chemicals in the world, H_2_O_2_ has been widely applied in the fields of organic synthesis, bleaching, disinfection, and environmental purification.^[^ ^1^, ^2^ ^]^ It is also a promising clean fuel with high energy density.^[^ ^3^ ^]^ With increasing market demand, the global yield of H_2_O_2_ is expected to reach ≈6 million tons by 2024.^[^ ^4^ ^]^ At present, the predominant technique for the production of H_2_O_2_ is through anthraquinone cycling process in industry.^[^ ^5^ ^]^ However, this process involves high energy consumption, a large amount of wastewater containing organic pollutions, and use of expensive catalysts.^[^ ^6^ ^]^ In addition, there are potential risks for concentrated solutions of H_2_O_2_ during the process of storage and transportation.^[^ ^7^ ^]^ Therefore, it is highly desirable to develop alternative techniques for green and on‐site production of H_2_O_2_.^[^ ^8^, ^9^, ^10^ ^]^

Photocatalytic ORR toward H_2_O_2_ production only needs sun, water, and oxygen, which represents a low‐cost, environmentally friendly, energy‐saving, and readily on‐site technique. Among various photocatalysts reported for H_2_O_2_ generation, RF resin photocatalysts have attracted much attention because of low cost, mature synthetic route, easy functionalization, and inert feature in the decomposition of H_2_O_2_.^[^ ^11^ ^]^ However, photocatalytic activity of the emerging photocatalysts is still unsatisfactory for practical applications. Several strategies have been used to improve photocatalytic ORR activity of RF resin such as increasing surface area,^[^ ^12^ ^]^ doping of conducting polymer,^[^ ^13^ ^]^ incorporation of phenol,^[^ ^14^ ^]^ and molecular modulation of 1,4‐dihydroxyanthraquinone.^[^ ^15^ ^]^ However, these methods often deal with multistep synthetic processes, additional introduction of organic substances, and the use of expensive raw materials.

Resorcinol used to synthesize RF resins contains two ─OH groups, which exhibited strong H‐bonding interactions.^[^ ^14^ ^]^ The existence of H‐bonding interactions probably affected *π*‐stacking of benzenoid (donor) and quinoid (acceptor) units within RF resin matrices, which can lead to decreased conductivity.^[^ ^16^ ^]^ On the other hand, photocatalytic ORR activity is closely associated with O_2_ adsorption ability. It has been reported that the introduction of S into carbon materials could increase their O_2_ adsorption ability.^[^ ^17^ ^]^ In addition, S‐containing organic building blocks can also adjust active centers to enhance ORR activity.^[^ ^18^ ^]^

Bearing the above in mind, herein, we synthesized 3‐HTPF resin photocatalysts through using 3‐HTP to replace resorcinol. It was found that the substitution of ─OH group by ─SH one could reduce H‐bonding interactions and strengthen donor–acceptor (D–A) *π*‐stacking, which increases crystallinity of the photocatalysts and forms stronger built‐in electric field and thus improves the separation of photogenerated electrons and holes. In addition, both O_2_ adsorption ability and ORR reaction activities can also be boosted due to the introduction of ─SH group. As a result, the as‐synthesized 3‐HTPF microspheres showed enhanced photocatalytic ORR activity toward H_2_O_2_ production because of collective effects of facile separation of photogenerated carriers, better adsorption ability to O_2_ molecules, and increased surface catalytic activity. Our work demonstrated a new strategy for designing highly efficient polymer photocatalysts via substituent group engineering.

## 2 Results and Discussion

**Figure 1:**
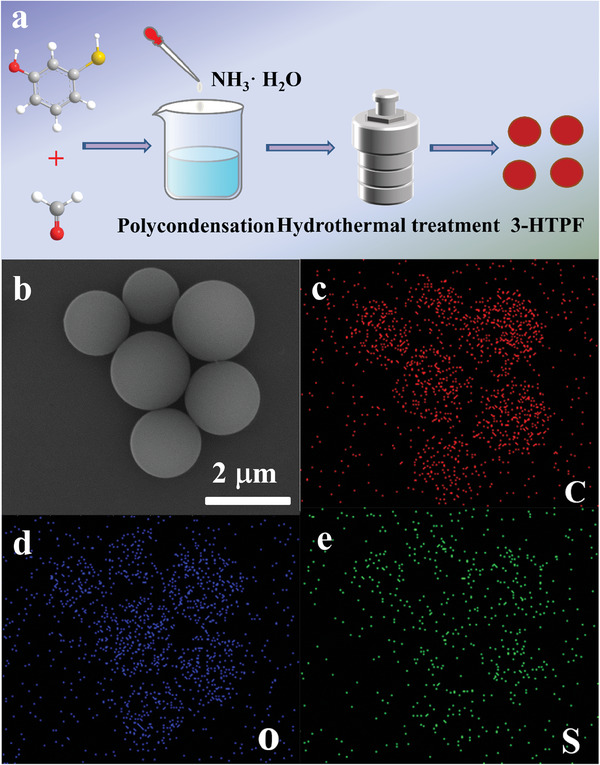
Schematical illustration of 3‐HTPF resin synthetic process (a). FE–SEM image of the as‐synthesized 3‐HTPF resin (b) and corresponding elemental mappings: C (c), O (d), and S (e).

3‐HTPF resin photocatalysts were synthesized through polycondensation reaction between 3‐HTP and formaldehyde in the presence of ammonia as a base catalyst at room temperature, followed by subsequent hydrothermal treatment at 250 °C, illustrated schematically in **Figure** **1**a. It was observed that the reaction solution for synthesis of RF became white immediately at room temperature, while it needed a longer time for the same color change for the reaction solution of 3‐HTPF, which indicates a slower polycondensation rate between 3‐HTP and formaldehyde than that of resorcinol and formaldehyde. 3‐HTP molecule has lower reaction activity than resorcinol because one of two ─OH groups in resorcinol molecule is replaced by another ─SH group. The electronegativity of S (χ = 2.5) is smaller than that of O (χ = 3.5),^[^ ^19^ ^]^ which can endow C reaction sites in 3‐HTP with fewer negative charges (Figure S1, Supporting Information). As a result, the nucleophilic addition of 3‐HTP to the highly electrophilic HCHO and quinone methides is more difficult than RF.^[^ ^11^, ^20^ ^]^ Usually, slow nucleation rate produces a small number of nuclei consuming the same amount of monomer and leads to large particles.^[^ ^21^ ^]^ As seen from Figure 1b, 3‐HTPF resin photocatalysts consist of microspheres with an average diameter of ≈1.7 µm, which is larger than that of RF submicrospheres (≈0.5 µm, Figure S2, Supporting Information). The corresponding energy‐dispersive X‐ray (EDX) elemental mappings (Figure 1c–e) show that the as‐synthesized 3‐HTPF microspheres are composed of C, O, and S elements, which supports that ─SH group is introduced into the resin.

**Figure 2:**
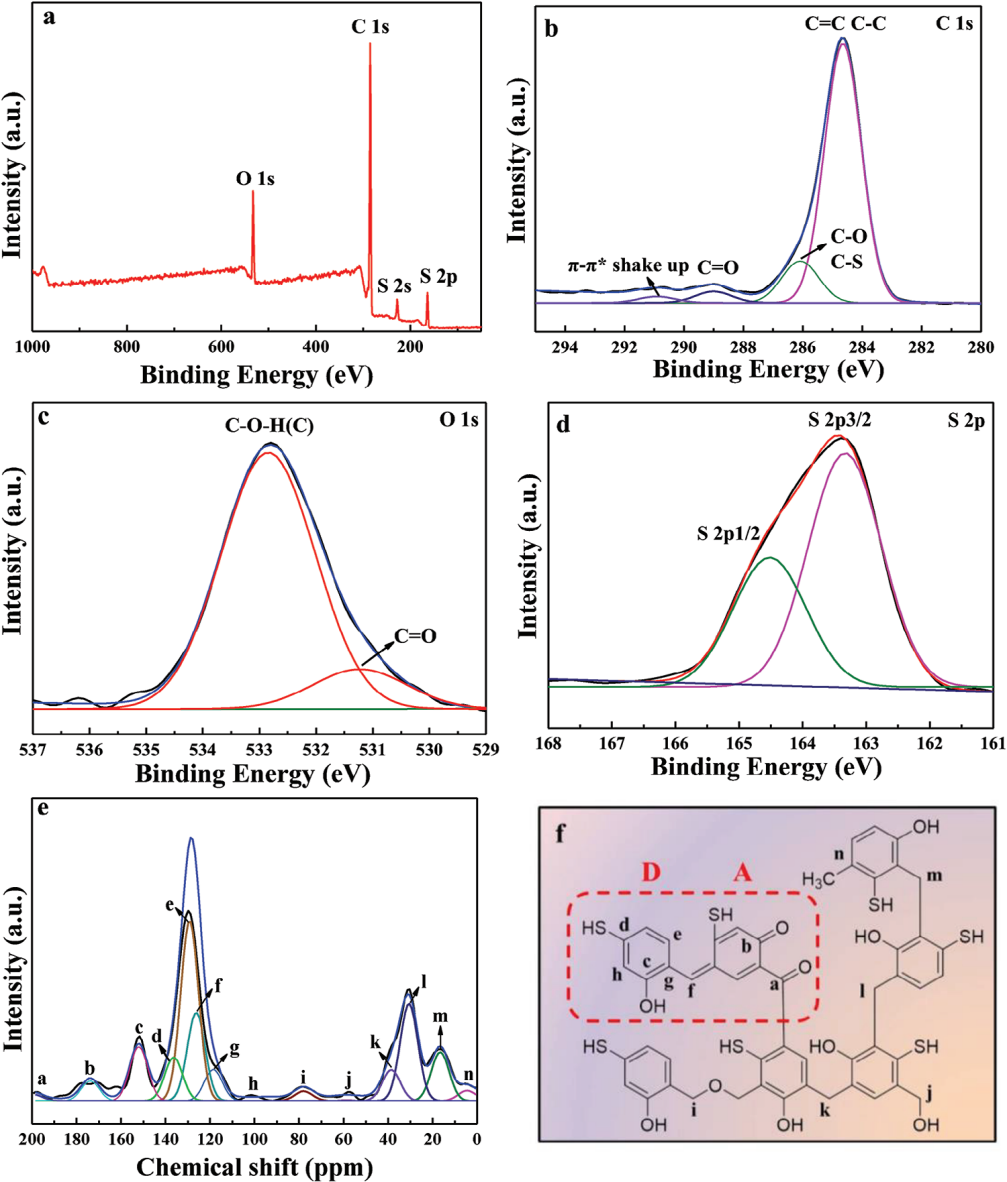
XPS survey spectrum (a) and high‐resolution C 1s (b), O 1s (c), and S 2p (d) of 3‐HTPF resin. Solid‐state ^13^C NMR spectrum of 3‐HTPF (e) and corresponding carbon structures in 3‐HTPF (f).

Compared with Fourier transform infrared (FT–IR) spectroscopy of RF, 3‐HTPF microspheres showed additional weak, wide S─H stretching vibration, C─S stretching vibration of Ar─S except for stretching vibrations from C═O, C─O, C─H, and C═C stretching vibration of the aromatic ring,^[^ ^22^, ^23^ ^]^ which further confirms that the ─SH group was introduced into the 3‐HTPF resin (Figure S3, Supporting Information). The analytical results of X‐ray photoelectron spectroscopy (XPS) are also consistent with those of FT–IRand EDX spectra. As seen from **Figure** **2**a, XPS survey spectrum shows the presence of C, O, and S in 3‐HTPF microspheres. High‐resolution XPS spectrum of C 1s can be deconvoluted into three main peaks located at 284.6, 286.1, and 288.8 eV (Figure 2b), which corresponds to C═C (C─C), C─O (S), and C═O bonds,^[^ ^24^ ^]^ respectively. For high‐resolution O 1s spectrum (Figure 2c), it can be divided into two peaks at 531.1 eV for quinone (C═O) and 532.8 eV for hydroquinone (C─O─H).^[^ ^11^ ^]^ As to S 2p spectrum (Figure 2d), the divided two peaks with gap of 1.2 eV (2p3/2 at 163.3 eV, 2p1/2 at 164.5 eV) both correspond to C─S─H bond.^[^ ^24^ ^]^ FT–IR spectrum, XPS spectrum, combined with previous studies indicate that benzenoid (D) and quinone (A) forms exist in 3‐HTPF resin.^[^ ^11^, ^14^, ^15^ ^]^ In order to further confirm this point, the solid‐state ^13^C nuclear magnetic resonance (NMR) spectrum of the resin was measured (Figure 2e). The ^13^C NMR spectrum of 3‐HTPF resin can be deconvoluted into 14 components (Figure 2f), assigned to ketone C═O (199 ppm, a), quinone C═O (174 ppm, b), C─OH in 3‐HTP (152 ppm, c), C─SH in 3‐HTP (136 ppm, d), nonsubstituted C at meta position (129 ppm, e), methine linker ─C═ (126 ppm, f), substituted C (118 ppm, g), nonsubstituted C (102 ppm, h), methylene ether linker ─C─O─C─ (78 ppm, i), methylol C─OH (59 ppm, j), and methylene linker ─C─ substituted to different positions of 3‐HTP (38 ppm, k; 30 ppm, l, 16 ppm, m), methyl CH_3_─ (5 ppm, n), respectively. The proposed carbon structures contain a D–A unit (Figure 2f). The cross‐linking degree was further evaluated through the ratio of the number of linker carbons to the number of aromatic rings.^[^ ^16^ ^]^ Compared with RF resin, 3‐HTP resin has a lower cross‐linking degree (Figure S4 and Tables S1 and S2, Supporting Information), namely, higher structural flexibility. This result means that D–A *π*‐stacking is easier to be achieved over the latter. Based on the above characterizations, it can conclude that DA typed 3‐HTPF resin microspheres with strongly *π*‐stacked architecture have been successfully synthesized.

**Figure 3:**
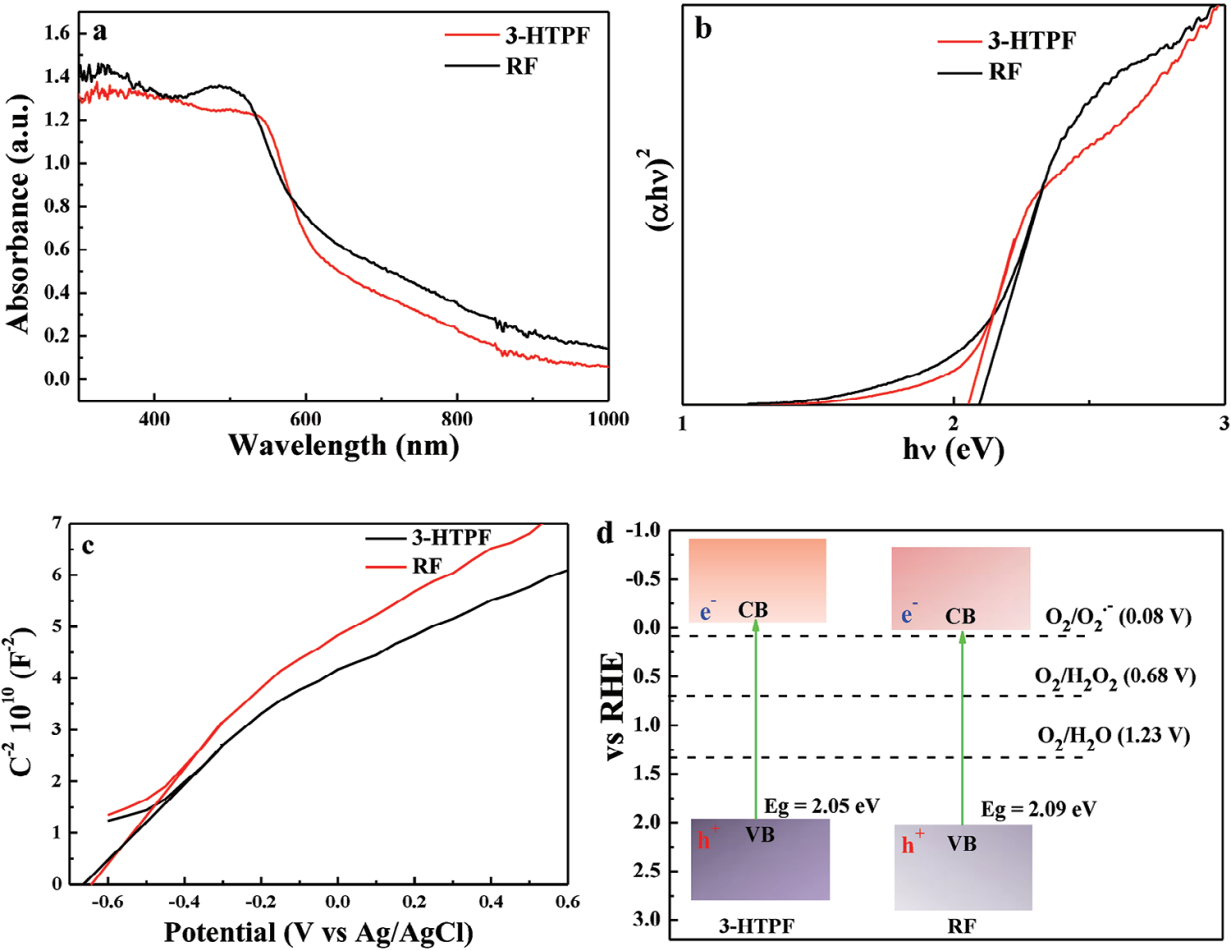
UV–vis absorption spectra (a), (ahυ)2 versus hυ curves (b), and Mott–Schottky curves of 3‐HTPF and RF resins (c). Schematical illustration of energy band positions of 3‐HTPF and RF resins and redox potentials for ORR and water oxidation toward H2O2 and O2, respectively (d).

The diffuse reflection UV–vis absorption spectra of 3‐HTPF and RF are shown in **Figure** **3**a. The spectral responsive range of 3‐HTPF microspheres is similar to that of RF submicrospheres. The bandgap energies of 3‐HTPF and RF estimated from their (ahυ)^2^ versus hυ curves are ≈2.05 and 2.09 eV, respectively (Figure 3b). 3‐HTPF has smaller bandgap energy than that of RF, which also supports the conclusion that the former has stronger *π*‐stacked D–A architecture owing to the higher structural flexibility. Figure 3c represents Mott–Schottky phots of 3‐HTPF resin and RF resin. Both of them show positive slopes, indicating their n‐type semiconductor feature.^[^ ^25^ ^]^ According to the Mott–Schottky equation

(1) $$\frac{1}{C^{2}}=\frac{2}{\varepsilon eNA^{2}}\;\left(E-E_{fb}\right)$$

where *C*, *ɛ*, *e*, *N*, *A*, *E*, and *E*_fb_ represent charge layer capacitance, relative dielectric constant of the sample, electron charge, donor density, surface area, applied potential, and flat potential, respectively. Smaller slope in the Mott–Schottky plot for 3‐HTPF resin means its larger carrier density than RF resin.^[^ ^26^ ^]^ The increased carrier density of 3‐HTPF probably comes from extended D–A *π*‐stacking. The order degree of aromatic D–A *π*‐stacking can be further reflected by XRD pattern and Raman spectrum. 3‐HTPF resin shows a sharper X‐ray diffraction (XRD) peak located at 2*θ* = 22.3^o^ assigned to (002) planes of graphitic carbon (Figure S5, Supporting Information), indicating that more organized *π*‐stacking between the D and A units in 3‐HTPF resin was formed than that in RF resin.^[^ ^14^ ^]^ Raman spectrum of 3‐HTPF show larger ratio of the intensities of the graphite G peak to the disorder D peak than that of RF (Figure S6, Supporting Information),^[^ ^27^ ^]^ which also shows more ordered *π*‐stacking of D–A units in 3‐HTPF than that in RF. The flat potentials derived from the intersections are −0.66 and −0.64 V versus Ag/AgCl for 3‐HTPF and RF, respectively. Considering that their flat potentials are close to conduction band (CB) bottoms, their CB bottoms are −0.07 and −0.05 V versus reversible hydrogen electrode (RHE) after they are converted into RHE. Correspondingly, their valence band positions are 1.98 and 2.04 V versus RHE, respectively. Therefore, as seen from Figure 3d, 3‐HTPF with a more negative CB position has a higher ORR ability toward H_2_O_2_ production. It is thermodynamically favorable for H_2_O_2_ production over 3‐HTPF resins through both direct concerted two‐electron ORR process (O_2_ + 2H^+^ + 2e^−^ → H_2_O_2_) and sequential two‐step single‐electron ORR process (O_2_ + e^−^_CB_ → O_2_^·−^, O_2_^·−^ + H^+^ → ·OOH, ·OOH + H^+^ + e^−^_CB_ → H_2_O_2_).^[^ ^28^ ^]^ In order to confirm this point, p‐benzoquinone as scavenger of superoxide radical was added into aqueous solution of photocatalytic ORR. As a result, H_2_O_2_ concentration obviously decreased, as shown in Figure S7 (Supporting Information). In addition, VB position of 3‐HTPF satisfies the thermodynamic conditions for oxidizing water to produce O_2_.

**Figure 4:**
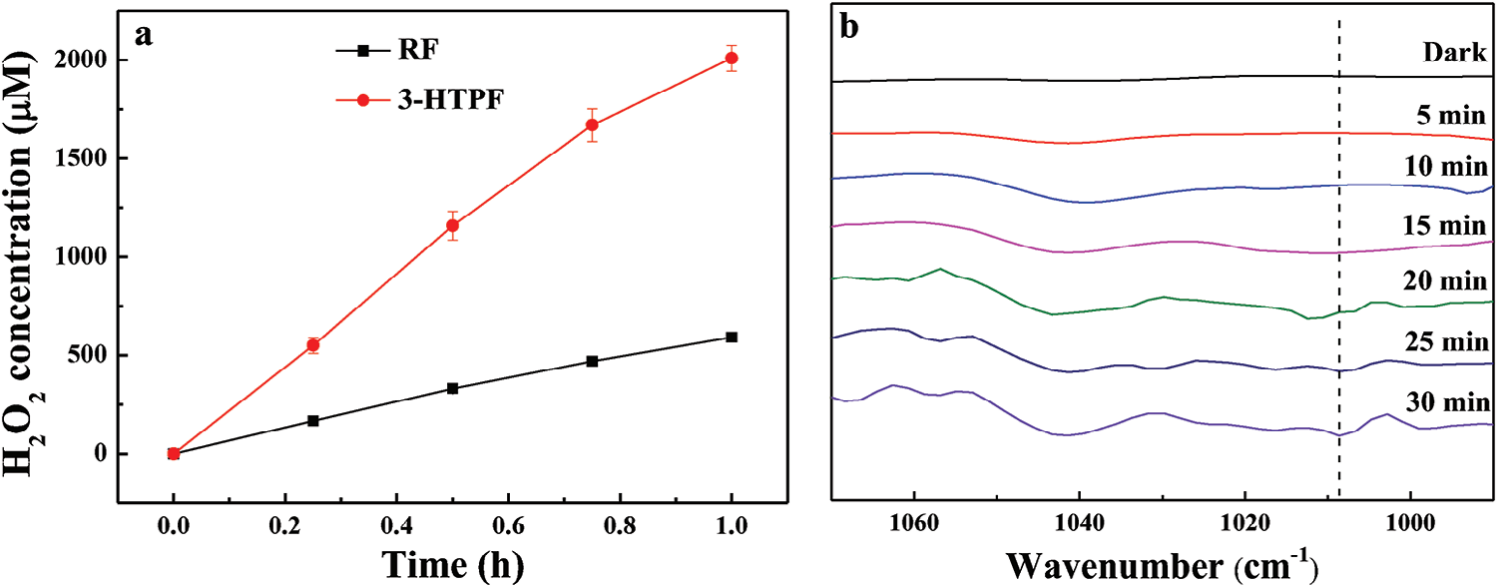
Visible light‐driven photocatalytic formation of H_2_O_2_ over 3‐HTPF and RF within 1 h (a). In situ DRIFT spectrum of 3‐HTPF in O_2_‐saturated water under visible light irradiation (*λ* > 420 nm; b).

The as‐obtained 3‐HTPF resin showed a significant higher visible‐light‐responding photocatalytic ORR activity toward H_2_O_2_ production than RF resin (**Figure** **4**a). Specifically, H_2_O_2_ production rate for 3‐HTPF resin is 2010 µm h^−1^ at 273 K, which is 3.4 times larger than that obtained on RF resin (591 µm h^−1^). If the reaction was carried out in the dark, no H_2_O_2_ was detected. When NaIO_3_ was used as an electron accepter in Ar‐saturated aqueous solution, only O_2_ and negligible H_2_O_2_ were produced. The above‐mentioned results show that H_2_O_2_ was produced through photocatalytic ORR process rather than chemical catalytic process.^[^ ^29^, ^30^ ^]^ In situ diffuse reflectance infrared Fourier transform (DRIFT) spectroscopy could further confirm the photocatalytic synthesis of H_2_O_2_ on the 3‐HTPF resin. As shown in Figure 4b, a new vibrational peak at 1008 cm^−1^ corresponding to the characteristic HOO stretching vibration of H_2_O_2_ was observed under visible light irradiation.^[^ ^31^ ^]^ With prolonged light irradiation time, the intensity of the vibration peak gradually increased, confirming the production of H_2_O_2_ over 3‐HTPF resin through photocatalytic ORR process. Although the rate of H_2_O_2_ production for 3‐HTPF resin somewhat decreased at initial reaction stage, it became almost unchanged after 5 h (Figure S8, Supporting Information), which shows long‐time photostability of the resin. In addition, the morphology, crystallinity, functional groups, and chemical states of the resin photocatalysts after ORR reaction had no obvious change (Figures S9–S12, Supporting Information), which also further confirmed their good stability. The apparent quantum yield of the as‐synthesized 3‐HTPF can reach 5.2% at 273 K. Photocatalytic ORR activity toward H_2_O_2_ production is among the best results (Table S3, Supporting Information).

**Figure 5:**
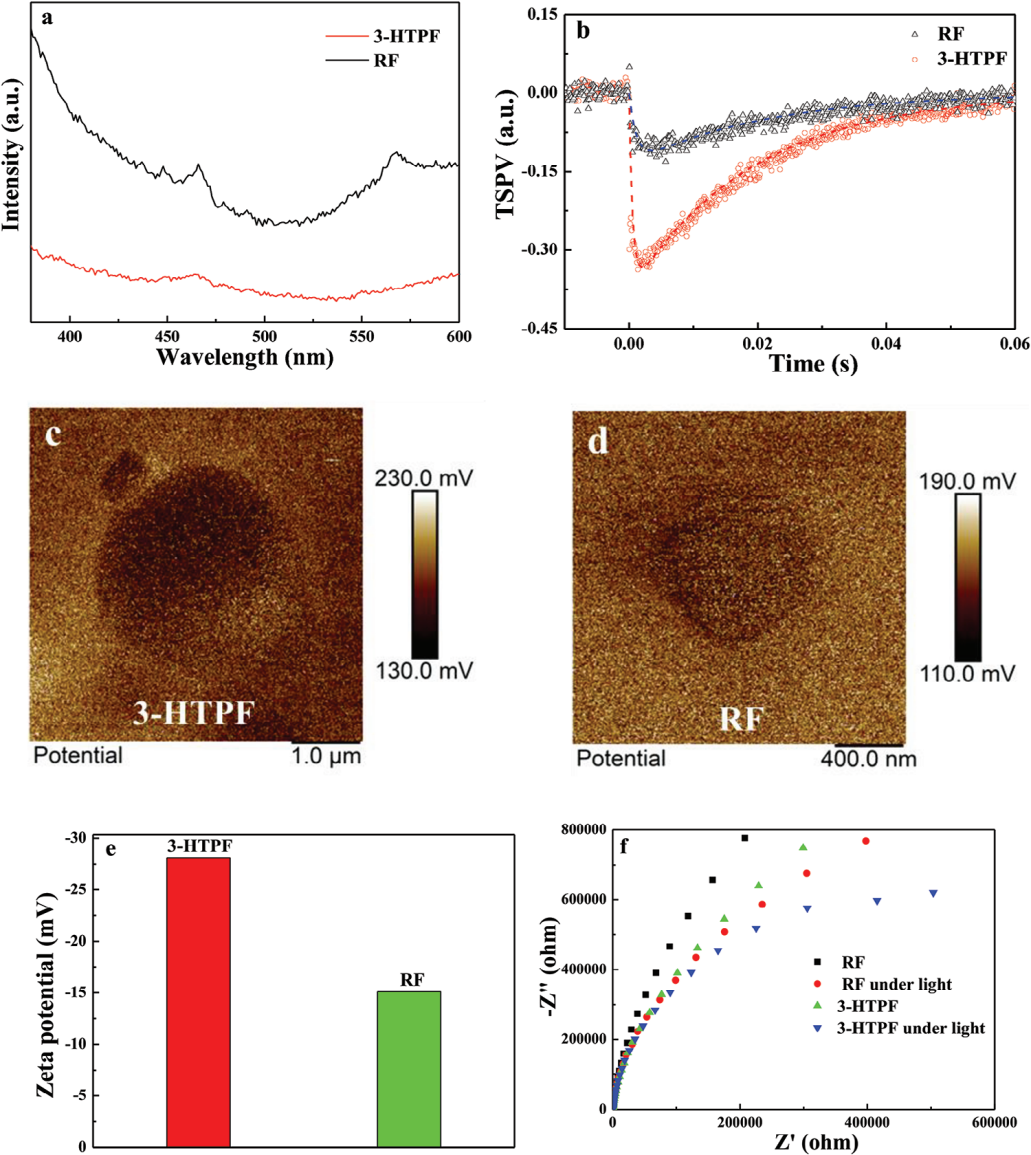
PL spectra (a), TSPV spectra (b), surface potential images (c,d), Zeta potential (e), and the impedance spectra of 3‐HTPF and RF resins (f).

The Brunauer–Emmett–Teller (BET) surface area of 3‐HTPF microspheres (1.1 m^2^ g^−1^) is much smaller than that of RF (64.8 m^2^ g^−1^), as calculated from N_2_ isotherms (Figure S13, Supporting Information), while the former shows higher photocatalytic activity than the latter. Considering that the spectral responsive range of 3‐HTPF microspheres is similar to that of RF (Figure 3a), therefore, 3‐HTPF resin possesses higher intrinsic photocatalytic activity than RF resin. In order to further ascertain the mechanism of the enhanced photocatalytic ORR activity of 3‐HTPF resin microspheres, their separation efficiency of photogenerated carriers, interfacial charge transfer resistance, O_2_ adsorption ability, surface catalytic activity was compared with those of RF resin submicrospheres. To compare separation ability of photogenerated carriers of 3‐HTPF‐and RF resin, their photoluminescence (PL) spectroscopy, and transient surface photovoltage spectroscopy (TSPV) were studied. PL intensity of the material is closely related to the recombination of photogenerated carriers. As shown in **Figure** **5**a, RF resin exhibits two PL peaks located at 466 and 568 nm, which is consistent with previous studies.^[^ ^15^, ^32^ ^]^ PL intensity of 3‐HTPF is lower than that of RF, which means that more photogenerated carriers were efficiently separated over the former than the latter. Figure 5b represents typical TSPV spectra of 3‐HTPF and RF resin. Both 3‐HTPFand RF resin exhibited negative signals of TSPV, proving the presence of surface electron acceptors (quinoid forms of resorcinol).^[^ ^12^ ^]^ Compared with RF resin, 3‐HTPF resin showed a larger photovoltage, which means that more photogenerated carriers were efficiently separated. According to our previous work,^[^ ^33^ ^]^ the transient accumulated charge (*Q_sep_*) versus the time (*t*) can be expressed as follows:

(2) $$Q_{sep}=\frac{k_{sep}Q_{exc,0}}{k_{cons}-k_{sep}}\;\left(e^{-k_{sep}t}-e^{-k_{cons}t}\right)$$

where *k*_sep_, *k*_cons_, *Q*_sep_, and *Q*_exc,0_ represent the rate constant for charge separation, charge surface reaction, surface accumulated charge, and the apparent initial surface charge, respectively. Based on Equation (2), *k_sep_* of 3‐HTPF and RF resin can be obtained through fitting the TSPV curves. As a result, *k_sep_* of 3‐HTP is 1800 and is 880 s^−1^ for RF. Therefore, the separation rate of photogenerated carriers of 3‐HTPF resin is faster than that of RF resin. Both PL and TSPV spectra showed that 3‐HTPF resin exhibited better separation ability of photogenerated electron and hole than RF resin.

The high charge separation ability of photogenerated carriers of 3‐HTPF resin can be attributed to its large built‐in electric field. According to previous study,^[^ ^34^ ^]^ the built‐in electric field is a monotonic function of its surface potentialand zeta potential for a given material. The surface potential can be determined by atomic force microscope with a Kelvin probe (KPFM). Figure 5c,d represents KPFM images of 3‐HTPF and RF. The average surface potential of 3‐HTPF resin is 176 mV, which is larger than that of RF (152 mV). On the other hand, as shown in Figure 5e, the Zeta potentials of 3‐HTPF (−28.1 mV) is larger than that of RF (−15.1 mV). 3‐HTPF resin exhibits both higher surface potentialand Zeta potential than RF. Therefore, 3‐HTPF resin has a larger built‐in electric field than RF, which can more efficiently promote the separation of photogenerated carriers. In addition, 3‐HTPF showed a larger photocurrentand smaller onset potential than those of RF under the same conditions (Figure S14, Supporting Information), which also support that 3‐HTPF possesses larger built‐in electric field. Furthermore, organic materials with larger molecular dipole can exhibit stronger built‐in electric field. Considering that 3‐HTPF has more organized *π*‐stacking between the Dand‐A units than that in RF resin, two possible 3‐HTPF‐and RF structure models were fabricated (Figure S15, Supporting Information). As a result, 3‐HTPF shows larger dipoles than RF in both cases. The simulation results also support that 3‐HTPF has larger built‐in electric field than RF. 3‐HTPF resin also showed smaller interfacial charge transfer resistance except for higher separation efficiency of photogenerated carriers. As shown in Figure 5f, the Nyquist plots of 3‐HTPF resin has a smaller impedance arc diameter than that of RF resin, indicating that faster charge transfer kinetics was achieved over the former. The decreased interfacial charge transfer resistance of 3‐HTPF mainly originated from its larger carrier densityand better conductivity caused by strongly π‐stacked architecture.

**Figure 6:**
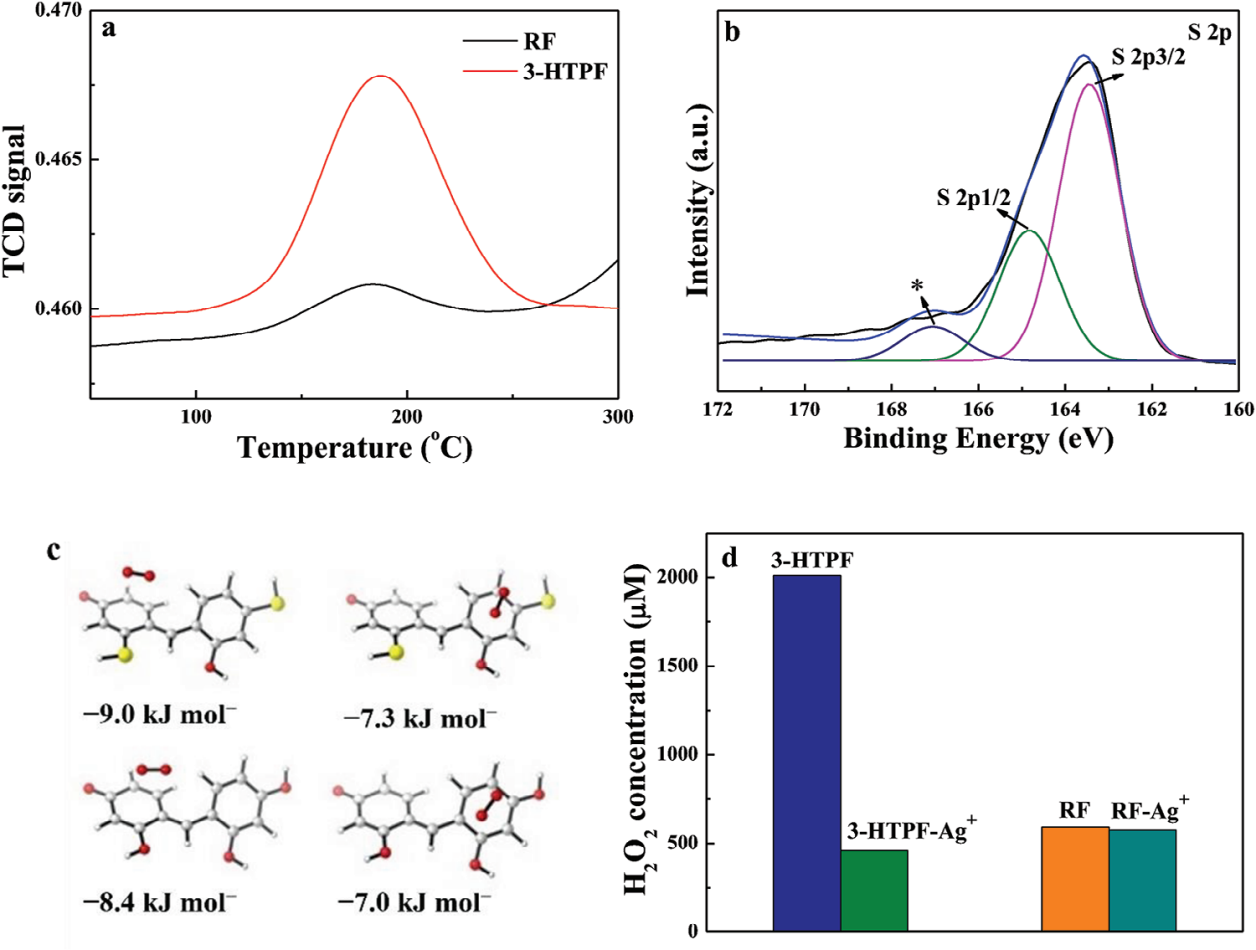
O_2_‐TPD profiles of 3‐HTPF and RF resins (a). High‐resolution S 2p XPS spectrum of 3‐HTPF after ORR (b). Adsorption energies of O_2_ on 3‐HTPF and RF resins (c). Photocatalytic ORR activity toward H_2_O_2_ production over 3‐HTPF and RF in the presence of Ag+ ions (d).

Since O_2_ adsorption on the photocatalyst is a prerequisite for photocatalytic ORR, the temperature programmed desorption (TPD)and adsorption isotherms of O_2_ adsorbed on 3‐HTPFand RF resins were measured (**Figure** **6**a; Figure S16, Supporting Information). Compared with RF, O2 TPD of 3‐HTPF exhibited stronger signals at relatively higher temperature (188 °C), showing its better O_2_ adsorption capacity. In addition, 3‐HTPF attained a O_2_ adsorption capacity of 435 cm^−3^ g^−1^ at 298 K, 1 atm, roughly 1.2 times larger than that of RF (358 cm^−3^ g^−1^). The enhanced O_2_ adsorption ability for 3‐HTPF can be ascribed to the introduction of ─SH group, which changed the surface chemistry of the resin, electrostatic interactions between O_2‐and_ the resin. As is expected, the atomic charge distribution of 3‐HTPF model molecule on the basis of natural population analysis (NPA) is distinct from that of RF one (Figure S17, Supporting Information), which means that the electrostatic interaction ability between 3‐HTPFandO_2_ is different from that between RFand O_2_. In addition, the ─SH groups in 3‐HTPF resin exhibit positive charges, suggesting that they could easily attract O_2_ molecules.^[^ ^35^ ^]^ In addition, XPS spectrum of 3‐HTPF after 1 h of reaction was investigatedand could further support this point (Figure S18, Supporting Information). It was observed that a new peak (marked by *) occurred in high‐resolution S 2p XPS spectrum of 3‐HTPF (Figure 6b). The corresponding binding energy of the peak (167.0 eV) is smaller than those of C─SO_2_ (168.2 eV) and C─SO_3_ (169.4 eV),^[^ ^36^, ^37^ ^]^ but larger than that of C─SH. The results showed that O_2_ was chemically adsorbed on ─SH groups in 3‐HTPF. Furthermore, the adsorption energies of O_2_ molecules on 3‐HTPFand RF molecular models at different positions were calculated. As shown in Figure 6c, the adsorption energies of O_2_ molecules on 3‐HTPF resin are lower than those of RF one, which is consistent with the higher O_2_ adsorption capability of 3‐HTPF resin than RF one.

As shown in Figure S17 (Supporting Information), corresponding atomic charge distribution in the resin was changed after one ─OH group was replaced by another ─SH group. In order to survey the influence of ─SH group on photocatalytic ORR, a catalyst poisoning test was carried out, using Ag^+^ ions as poisoning reagent due to their strong complexing ability.^[^ ^38^ ^]^ As shown in Figure 6d, photocatalytic ORR activity of 3‐HTPF after adsorption of Ag^+^ ions toward H_2_O_2_ production obviously decreased, while it had negligible change for RF under the same conditions. The result shows that ─SH groups could modulate photocatalytic ORR active centers toward H_2_O_2_ generation and enhanced their activities. The electrochemical rotating ring‐disk electrode (RRDE) measurements also showed that 3‐HTPF resins have a larger ring current, meaning that more H_2_O_2_ was produced on the resins (Figure S19, Supporting Information). That is to say, 3‐HTPF possesses higher surface catalytic activity for ORR toward H_2_O_2_ production.

**Figure 7:**
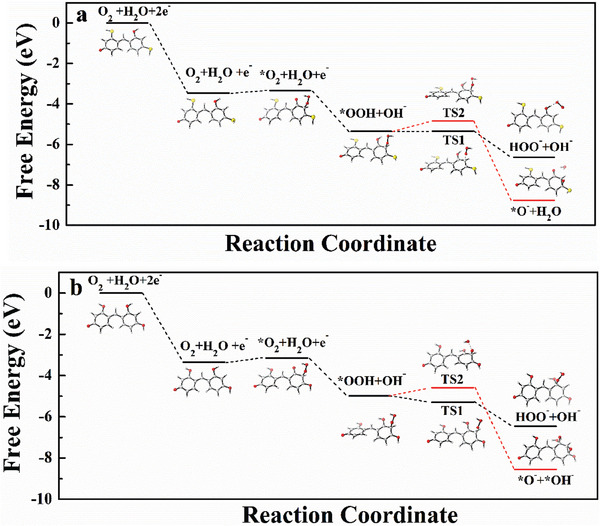
Energy profiles of key possible ORR pathways on 3‐HTPF (a) and RF (b) and corresponding optimized geometries for the reaction intermediates. Yellow, red, gray, and black balls represent sulfur, oxygen, hydrogen, and carbon, respectively.

In order to understand enhanced photocatalytic ORR activity of 3‐HTPF toward H_2_O_2_ production, density functional theory (DFT) calculations were carried out. **Figure** **7**a,b represents free energy diagrams of possible photocatalytic ORR pathways on 3‐HTPF and RF, respectively. The addition of an electron to 3‐HTPF and RF are both exothermic in an aqueous solution phase. Therefore, reduction of 3‐HTPF and RF via electron attachment to form the negative ions of 3‐HTPF and RF is regarded as the first step. At this step, more energy was released from 3‐HTPF (3.47 eV) than from RF (3.35 eV), indicating that 3‐HTPF more easily accepted an electron and formed negative ion of 3‐HTPF. The further calculation results based on structural model of 3‐HTPF anion (Figure S20, Supporting Information) show that O_2_ adsorption has the lowest Gibbs free energies when C2 of 3‐HTPF is used as the O_2_ adsorption site (Figure S21 and Table S4, Supporting Information). Thus, C2 was chosen as O_2_ adsorption site for subsequent conversion. Addition of an O_2_ molecule to the 3‐HTPF^−^ and RF^−^ anions leads to the formation of peroxy anions 3‐HTPF─O_2_^−^and RF─O_2_^−^, a step that is endothermic by 0.14 and 0.2 eV, respectively. Hence, 3‐HTPF^−^ anion is more favorable for O_2_ addition in energy. The peroxy anions abstracts a proton from a water molecule while accepting an electron in a proton‐coupled step to form 3‐HTPF–OOH + OH^−^ and RF–OOH + OH^−^, which is exothermic by 2.02 and 1.83 eV, respectively. The next step is the cleavage of C─OOH bond to evolve a peroxide ion HO_2_ (black lines) or the cleavage of CO─OH bond to evolve a OH^−^ ion. The OH^−^ ion will further capture active H in 3‐HTPF and formed H_2_O (red lines). C─OOH bond cleavage leads to the recovery of the starting 3‐HTPF and the formation of HOO^−^ ions. In contrast, CO─OH bond cleavage results in the generation of epoxide anion of 3‐HTPF═O, which requires further reduction to evolve the second water molecule. The two different bond cleavage involve 2e^−^ (H_2_O_2_) and 4e^−^ (H_2_O) ORR process, respectively.

To study the ORR selectivity toward H_2_O_2_‐and H_2_O over 3‐HTPFand RF, the transition state (TS) structures (Figures S22 and S23, Supporting Information)and TS theory were used. TS for the C─OOH and CO─OH bond cleavage steps were marked with TS1 and TS2, respectively. To 3‐HTPF, at the TS1 for C─OOH cleavage, the C─OOH distance is ≈1.711 Å while at the TS2 for CO─OH cleavage, the CO─OH distance is somewhat shorter ≈1.697 Å. This result indicates that C─OOH bond is easier to rupture than CO─OH bond with the calculated energy barriers of −0.01 and 0.51 eV for the cleavage of C─OOH and CO─OH, respectively. According to TS theory where the reaction rate *k* = Aexp(−ΔG*/*k*_B_ *T*) (ΔG* is the energy barrier), the reaction rate for HOO^−^ formation is ≈6 × 10^8^ times faster than the reaction rate for OH^−^ formation. Therefore, the 2e pathway is kinetically favored over the 4e pathway, leading to the high selectivity for H_2_O_2_ production. Similarly, the 2e pathway is kinetically favored over RF as well. However, more energy was released from TS1 to the formation of HOO^−^ anion for 3‐HTPF (1.3 eV) than RF (1.16 eV). Therefore, ORR toward H_2_O_2_ is more energetically favorable over 3‐HTPF than RF, the introduction of ─SH groups could thermodynamically promote ORR of 3‐HTPFand simultaneously keep high H_2_O_2_ production selectivity.

The enhanced photocatalytic ORR activity of 3‐HTPF resin toward H_2_O_2_ production can be at least ascribed to the following points: 1) The reaction activity of 3‐HTP for the nucleophilic addition to electrophilic HCHO and quinone methides was lower than that of resorcinol. The slow nucleation led to order *π*‐stacking of D–A units in 3‐HTPF resin and formed larger particles. Improved crystallinity induced larger built‐in electric field, which promoted the separation of photogenerated carriers; 2) The presence of ─SH group could boost O_2_ adsorption ability of the photocatalyst. 3) The ─SH group modulated atomic charge distribution in 3‐HTPF molecules, leading to the formation of H_2_O_2_ are both thermodynamically and kinetically favorable.

## 3 Conclusion

In summary, 3‐HTPF resin microspheres have been synthesized through a facile hydrothermal route. The as‐synthesized products showed highly efficient photocatalytic ORR activity for H_2_O_2_ generation. As a result, H_2_O_2_ production rate for 3‐HTPF resin could reach 2010 µm h^−1^ at 273 K under visible light irradiation, which is 3.4 times larger than that obtained over RF resin (591 µm h^−1^). The experimental results and theoretical calculations indicate that the introduction of ─SH group plays key roles in enhancing photocatalytic ORR activity toward H_2_O_2_ production. The enhanced photocatalytic ORR activity of 3‐HTPF resin could be attributed to its larger built‐in electric field, stronger adsorption ability to O_2_ molecules, and enhanced ORR activity. The present work not only provides a new polymer photocatalyst but also demonstrates the effectiveness of molecule engineering for modulating photocatalytic ORR activity.

## 4 Experimental Section

### Chemicals and Materials

3‐HTP, resorcinol, polyformaldehyde, potassium iodide, and potassium biphthalate were purchased from J&K Scientific. Ammonium molybdate tetrahydrate was obtained from Aladdin. Ammonia aqueous solution (NH_3_·H_2_O, 25–28 wt.%) was received from Hangzhou Banyao Heshun Chemical Reagent Factory. H_2_O_2_ was supplied by Sinopharm Chemical Reagent Factory. All chemicals were used as received without further purification.

### Syntheses of 3‐HTPF and RF Resins

For the synthesis of 3‐HTPF, in a typical process, 1 mmol of 3‐HTP, 2 mmol of polyformaldehyde, and 1 mmol of ammonia aqueous solution were added to 40 mL of distilled water. Then the solution was stirred for 10 min at room temperature. After that, it was transferred to a Teflon‐lined stainless‐steel autoclave and heated at 250 °C for 24 h. After the reaction was completed, the products were collected by centrifugation and washed with ethanol and ultrapure water for three times at least. The obtained solid products were dried in a vacuum drying oven at 60 °C for 6 h. The synthetic procedure of RF resins was similar to that of 3‐HTPF ones except that 3‐HTP was replaced by resorcinol.

### Photocatalytic H2O2 Production

Fifty milligrams of 3‐HTPF resins was ultrasonically dispersed in 30 mL ultrapure water. Then the solution was oxygen‐saturated by bubbling oxygen into the solution for 30 min under dark conditions, stirred at 100 rpm. A 300 W xenon lamp (PLS‐SXE300+, Perfectlight) with a 420 nm cutoff filter was used as the light source. The temperature of the reaction solution was kept at 273 K. The solution was continuously stirred during the reaction process. At certain intervals, 1 mL of the suspension was taken out, filtered through a 0.45 µm needle‐type filter. The concentration of H_2_O_2_ in solution was determined according to modified I_3_^−^ method.^[^ ^39^ ^]^ The detailed process is provided in the Supporting Information.

### Characterization

Field emission scanning electron microscopy (FE–SEM) images and elemental mapping were obtained from Nova NanoSEM 200. The infrared reflectance spectra of the samples were recorded on Fourier transform infrared spectrometer. Raman spectra were conducted on Thermo Fisher DXR3 Raman spectrometer and the laser wavelength is 523 nm. UV–vis absorption spectra were recorded on shimadzu UV‐2550, using BaSO_4_ as the reference. XPS measurement was carried out using a Thermo SCIENTIFIC ESCALAB 250Xi with Al K alph (hυ = 1486.8 eV) as the excitation source. The XPS data were calibrated with the C1s peak (284.8 eV). The XRD was carried out on a PANayltical Empyrean powder X‐ray diffractometer and the scan rate is 0.02° s^−1^. The photoluminescence spectrum was recorded on Fluoromax‐4 and the excitation wavelength was 370 nm. N_2_ and O_2_ adsorption/desorption isothermals were obtained from Quantachrome autosorb IQ and BELSORP MAX X, respectively. O_2_ temperature programmed desorption (O_2_‐TPD) measurement was carried out on Chembet Pulsar TPD using He as carrier gas. TSPV spectra of the samples were measured on FTO substrate in air at room temperature, excited with a Nd: YAG nanosecond laser (Quantel 450, 355 nm, 80 µJ per pulse) and low‐noise voltage preamplifier (5186 and SR560). The surface potential profiles were obtained by KPFM on an atomic force microscope (AFM, Bruker Dimension Icon) with a conductive probe (SCM–PIT).

### Electrochemical Measurements

All electrochemical measurements were carried out with a standard three‐electrode configuration on a CHI760E electrochemical working station, using the as‐synthesized samples coated at F‐doped FTO glass (1 cm × 2 cm) as the working electrode, a Pt wire as the counter electrode, and a saturated calomel electrode as the reference electrode. Electrochemical impendence spectra were measured under illumination and in the dark with an amplitude of 5 mV in the range of 10^5^ to 1 Hz, using 0.1 m Na_2_SO_4_ as electrolyte. The Mott–Schottky plots were measured at 1000 Hz within potential range from −0.6 to 0.8 V versus Ag/AgCl. The Zeta potentials of the samples were obtained on a Zeta potentiostat (Zetasizer Nano ZS90). RRDE measurements were performed on a Pine rotator system.

### Theoretical Calculations

DFT calculations were conducted with the Gaussian 16 program. The detailed calculation methods are provided in Supporting Information.

## Conflict of Interest

The authors declare no conflict of interest.

## Supporting information

Supporting Information

## Data Availability

The data that support the findings of this study are available from the corresponding author upon reasonable request.
