# Supplementary material for: 3‐Hydroxythiophenol‐Formaldehyde Resin Microspheres Modulated by Sulfhydryl Groups for Highly Efficient Photocatalytic Synthesis of H2O2
Source: Adv Sci (Weinh). 2023 Dec 10;11(37):2304948. doi: 10.1002/advs.202304948 (PMC11462293; doi:10.1002/advs.202304948)
Supplement: Supplementary file 1 — Supporting Information [file ADVS-11-2304948-s001.pdf]

ADVANCED SCIENCE
Open Access

Supporting Information

for *Adv. Sci.*, DOI 10.1002/advs.202304948

3-Hydroxythiophenol-Formaldehyde Resin Microspheres Modulated by Sulfhydryl Groups for Highly Efficient Photocatalytic Synthesis of H_2O_2

Yulu Xu, Xia Hu, Yuyuan Chen, Sijie Lin, Chen Wang*, Faliang Gou, Xiaogang Yang*, Weiwei Zheng and De-Kun Ma**

3-hydroxythiophenol-formaldehyde resin microspheres modulated by sulfhydryl groups for highly efficient photocatalytic synthesis of H_2O_2

Yulu Xu, Xia Hu,* Yuyuan Chen, Sijie Lin, Chen Wang,* Faliang Gou, Xiaogang Yang,* Weiwei Zheng, and De-Kun Ma*

Experimental section

H_2O_2 concentration detection method: The concentration of H_2O_2 was determined by I_3^- method. To be specific, 250 μL of filtered solution was taken out and diluted to 4 mL, followed by the addition of 0.1 mL of potassium iodide solution (0.4 M), 0.1 mL of potassium biphthalate solution (0.1 M), and 0.1 mL of ammonium molybdate solution (0.1 mM). The solution was shaken for 15 min. Then the absorbance of the solution at 350 nm (I_3^-) measured by UV-vis spectroscopy was recorded. The reaction mechanism is that H_2O_2 can oxidize KI to I_2 and I_2 is dissolved in KI to form I_3^-. I_3^- has absorbance at 350 nm. The absorbance of I_3^- is proportional to its concentration according to the Lambert-Beer law. Further, the concentration of H_2O_2 can be calculated based on the stoichiometric relationship of the reaction between H_2O_2 and KI.

Plotting of H_2O_2 standard curve: 50 μL of 30% H_2O_2 was taken out and diluted to 50 mL to get a concentration of 10 mM H_2O_2 solution. Taking 12, 20, 28, 36, 44 μL of 10 mM H_2O_2 solution diluted to 4 mL, respectively, to obtain H_2O_2 solutions with concentrations of 30, 50, 70, 90, 110 μM. Subsequently, 0.1 mL of potassium iodide solution (0.4 M), 0.1 mL of potassium biphthalate solution (0.1 M), and 0.1 mL of ammonium molybdate solution (0.1 mM) were added in the standard solution of H_2O_2, respectively. The standard calibration was obtained by measuring the absorbance at 350 nm of the above solutions.

Computational Methods

Density functional theory (DFT) calculations were conducted with the Gaussian 16 program.[1] Geometry optimization was performed using B3LYP functional [2] with def2-SVP basis set,[3] as well as Grimme's DFT dispersion correction with Becke-Johnson damping (DFT-D3(BJ)).[4] Frequency calculations were performed to confirm stationary points as minima or transition states using the same method with the optimizations. Single-point energy calculations in aqueous

solution were then performed on the stationary points by using M06-2X [5] functional with def2-TZVP basis set with SMD model.[6] Single-point energies corrected by Gibbs free energy corrections were used to as the solution phase Gibbs free energies. All the energies in this paper correspond to the reference state of 1 mol L^{-1}, 298.15 K. The natural population analysis (NPA) charges reported and the dipole moments are calculated at the B3LYP-GD3(BJ)/def2-SVP level. The 3-D structures were drawn using CYLview software.[7]

The adsorption energies (ΔE) of 3-HTPF and RF were calculated according to the equation below:

$\Delta E = E(\text{model-}O_2) - E(\text{model}) - E(O_2)$

Where E(model-O_2), E(model) and E(O_2) are the single-point energies of optimized model of adsorbed model, model and O_2, respectively.

References

[1] M. J. Frisch, G. W. Trucks, H. B. Schlegel, G. E. Scuseria, M. A. Robb, J. R. Cheeseman, G. Scalmani, V. Barone, G. A. Petersson, H. Nakatsuji, X. Li, M. Caricato, A. V. Marenich, J. Bloino, B. G .Janesko, R. Gomperts, B. Mennucci, H. P. Hratchian, J. V. Ortiz, A. F. Izmaylov, J. L. Sonnenberg, F. Ding, F. Lipparini, F. Egidi, J. Goings, B. Peng, A. Petrone, T. Henderson, D. Ranasinghe, V. G. Zakrzewski, J. Gao, N. Rega, G. Zheng, W. Liang, M. Hada, M. Ehara, K. Toyota, R .Fukuda, J. Hasegawa, M .Ishida, T. Nakajima, Y. Honda, O. Kitao, H. Nakai, T. Vreven, K. Throssell, Jr., J. A. Montgomery, J. E. Peralta, F. Ogliaro, M. J. Bearpark, J. J. Heyd, E. N. Brothers, K. N. Kudin, V. N. Staroverov, T. A. Keith, R. Kobayashi, J. Normand, K. Raghavachari, A. P. Rendell, J. C. Burant, S. S. Iyengar, J. Tomasi, M. Cossi, J. M. Millam, M. Klene, C. Adamo, R. Cammi, J. W. Ochterski, R. L. Martin, K. Morokuma, O. Farkas, J. B. Foresman, D. J. Fox, Gaussian 16 Rev. C.01, Wallingford, CT, 2016.

[2] (a) A. D. Becke, *Phys. Rev. A* **1988**, *38*, 3098; (b) C. Lee, W. Yang, R. G. Parr, *Phys. Rev. B* **1988**, *37*, 785.

[3] (a) F. Weigend, R. Ahlrichs, *Phys. Chem. Chem. Phys.* **2005**, *7*, 3297; (b) F. Weigend, *Phys. Chem. Chem. Phys.* **2006**, *8*, 1057.

[4] (a) S. Grimme, J. Antony, S. Ehrlich, H. Krieg, *J. Chem. Phys.* **2010**, *132*, 154104; (b) A. D. Becke, E. R. Johnson, *J. Chem. Phys.* **2005**, 123, 154101.

[5] (a) Y. Zhao, D. G. Truhlar, *J. Phys. Chem.* A. **2006**, *110*, 13126; (b) Y. Zhao, D. G. Truhlar, *Acc. Chem. Res*. **2008**, *41*, 157; (c) Y. Zhao, D. G. Truhlar, *Chem. Phys. Lett*. **2011**, *502*, 1.

[6] A. V. Marenich, C. J. Cramer, D. G. Truhlar, *J. Phys. Chem. B* **2009**, *113*, 6378.

[7] C. Y. Legault, CYLView, 1.0b; Universitéde Sherbrooke: Québec, Montreal, Canada, 2009; http://www.cylview.org.

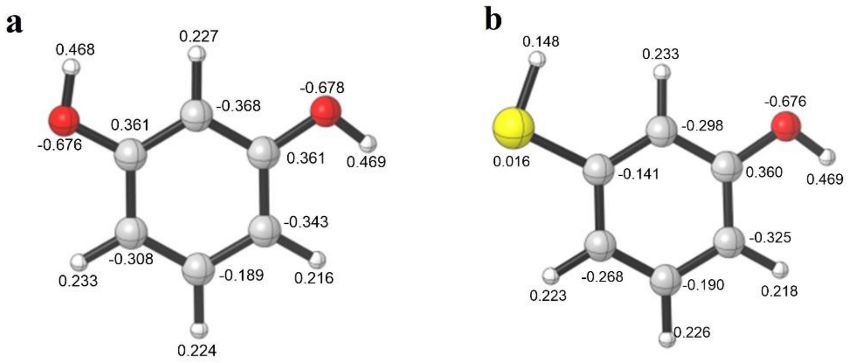

Figure S1. The atomic charges obtained from natural population analysis for RF (a) and 3-HTPF molecules (b).

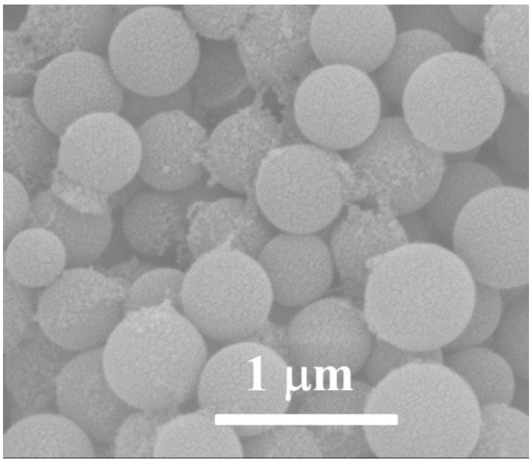

Figure S2. FE-SEM image of the as-synthesized RF.

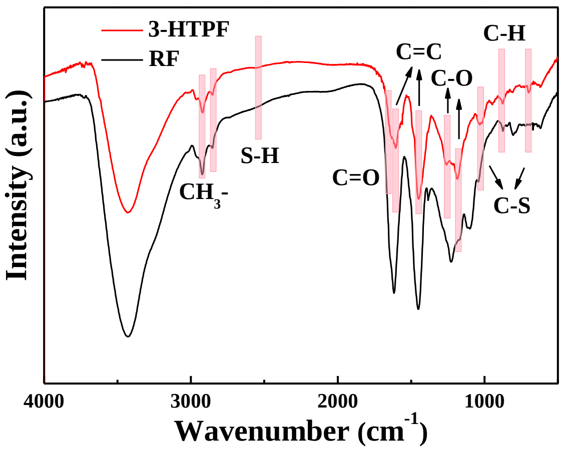

Figure S3. FT-IR spectra of 3-HTPF and RF resins.

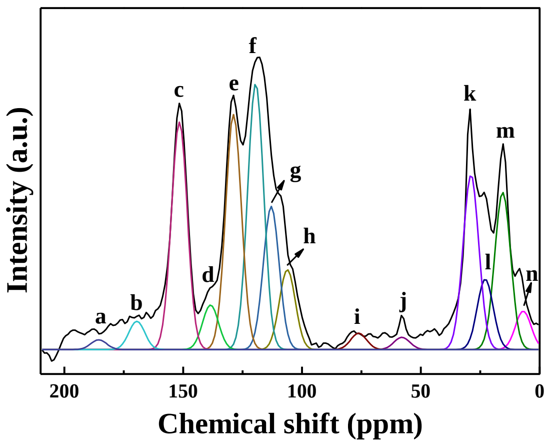

Figure S4. Solid-state ^{13}C NMR spectrum of RF resin.

Table S1. Carbon compositions of 3-HTPF resins

Resins	Aromatics (%) (b, c, d, e, g, h)	Linkers (%)				Residual groups (%)	
		Methylene ether (i)	Methylene (k, l, m)	Methine (f)	Ketone (a)	Methylol (j)	Methyl (n)
**3-HTPF**	53.44	1.55	28.12	14.02	1.02	0.22	1.63

Table S2. Carbon compositions of RF resins

Resins	Aromatics (%) (b, c, d, f, g, h)	Linkers (%)				Residual groups (%)	
		Methylene ether (i)	Methylene (k, l, m)	Methine (e)	Ketone (a)	Methylol (j)	Methyl (n)
**RF**	43.48	1.29	31.84	18.6	0.77	0.97	3.05

The ratio of the number of linker carbons to the number of aromatic rings was determined using the following equation

$$\text{linker/aromatic ring} = \frac{[all\ linker\ carbons]}{[all\ aromatic\ carbons]} \times 6$$

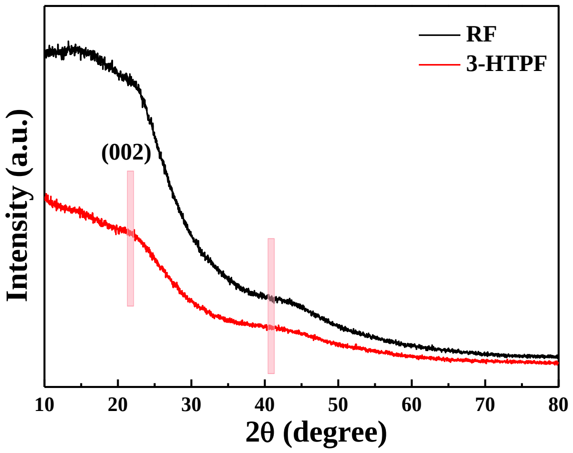

Figure S5. XRD patterns of 3-HTPF and RF resins.

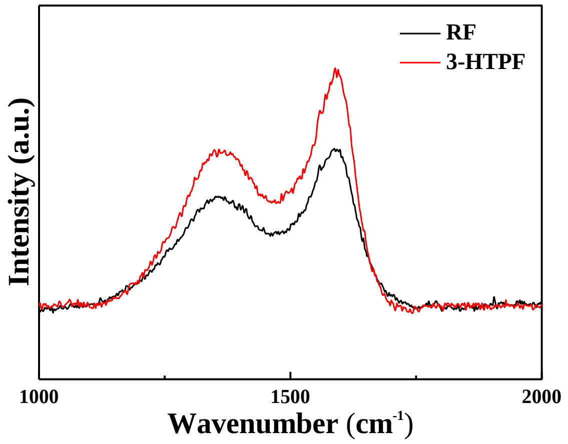

Figure S6. Raman spectra of 3-HTPF and RF resins.

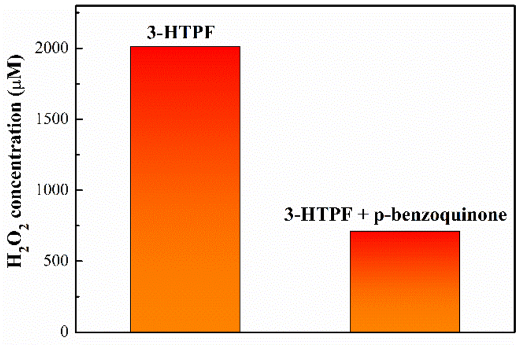

Figure S7. H_2O_2 concentration changes in the presence of p-benzoquinone.

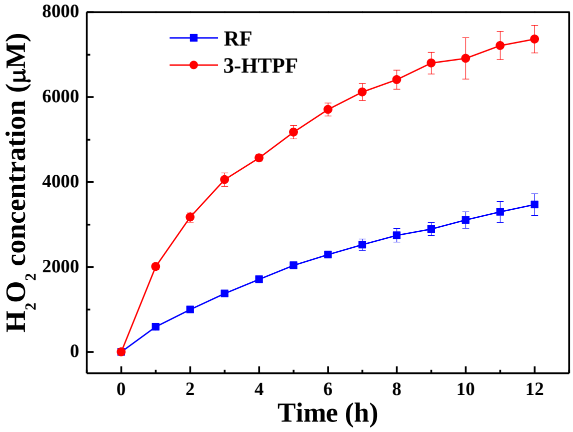

Figure S8. Visible light-driven photocatalytic formation of H_2O_2 over 3-HTPF and RF resins under long-time irradiation.

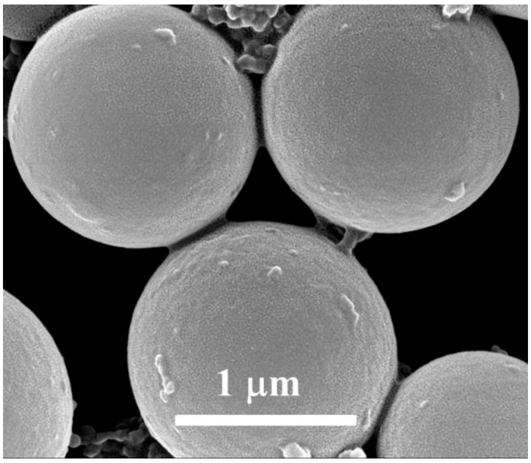

Figure S9. FE-SEM image of the sample after 12 hours of reaction.

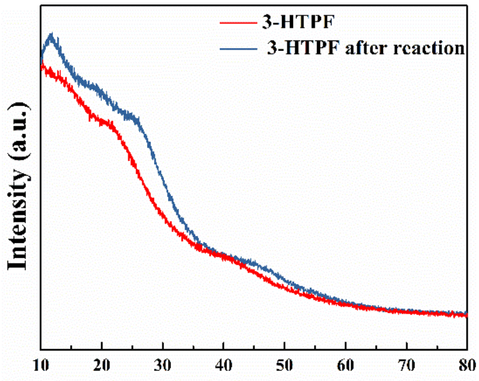

Figure 10. XRD patterns of pristine 3-HTPF and 3-HTPF resins after photocatalytic ORR.

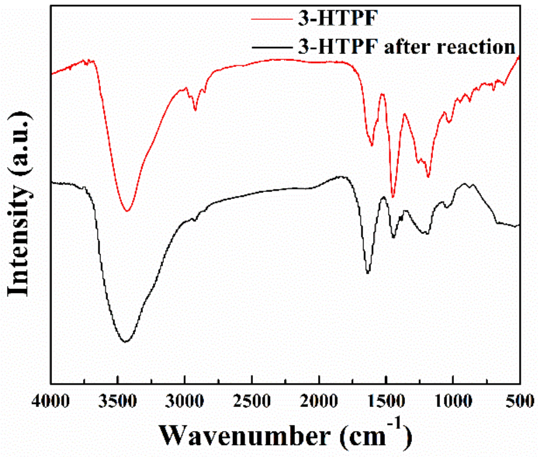

Figure 11. FTIR spectra of pristine 3-HTPF and 3-HTPF resins after photocatalytic ORR.

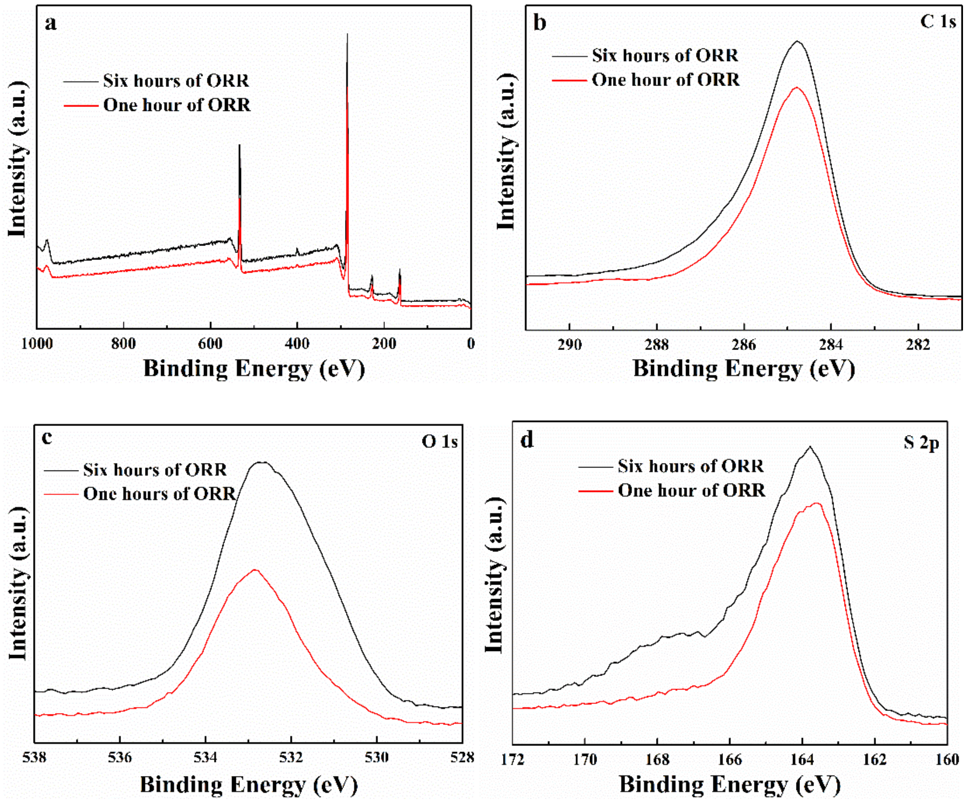

Figure 12. XPS survey spectra (a), high-resolution C1s (b), O 1s (c), and S 2p (d) spectra for pristine 3-HTPF and 3-HTPF resins after photocatalytic ORR.

Table S3. Comparison of photocatalytic activity of representative photocatalysts for ORR toward H_2O_2 production.

**Photocatalysts**	**Reaction medium**	**Dosage of catalyst**	**Irradition source**	**H_2O_2 production rate**	**Refs.**
TiO_2	2-propanol	5 g L^{-1}	365 nm	83×10^{-6} M min^{-1}	1
ZnO	Ethanol	0.5 g L^{-1}	300 W Xe lamp (350 nm ≤ λ ≤ 780 nm)	570 μmmol g^{-1} h^{-1}	2
$ZnIn_2S_4$	2-propanol + water	0.5 g L^{-1}	300 W Xe lamp (λ≥420 nm)	1592.04 μmol g^{-1} h^{-1}	3
Ultrathin g-C_3N_4	Ethanol+water	1 g L^{-1}	Simulated AM 1.5G	1083 μmol g^{-1} h^{-1}	4
Sb-SAPC15	O_2-saturated water	2 g L^{-1}	300 W Xe lamp (λ>420 nm)	235 μmol g^{-1} h^{-1}	5
RF523	O_2-saturated water	1.67 g L^{-1}	λ≥420 nm	2.58 μmol h^{-1}	6
RF/P3HT-1.0	O_2-saturated water	1.67 g L^{-1}	λ≥420 nm	11.6 μmol h^{-1}	7
RF-acid-resins	O_2-saturated water	1.67 g L^{-1}	2000 W Xe lamp (λ>420 nm)	100 μmol h^{-1}	8
Mesoporous resin nanobowls	O_2-saturated	1.67 g	300 W	19.4 mM g^{-1} h^{-1}	9

	water	L^{-1}	Xe lamp (λ>420 nm)		
Benzoxazine-based APFac	O_2-saturated water	1.67 g L^{-1}	300 W Xe lamp (λ>420 nm)	11.3 μmol h^{-1}	10
hydroxythiophenol-formaldehyde resin	O_2-saturated water	1.67 g L^{-1}	300 W Xe lamp (λ>420 nm)	2010 μM h^{-1}	This work

References

[1] B. O. Burek, D. W. Bahnemann, J. Z. Bloh, *ACS Catal*. **2019**, *9*, 25.

[2] Z. Jiang, Y. Zhang, L. Zhang, B. Cheng, L. Wang, *Chin. J. Catal.* **2022,** *43*, 226.

[3] K. L. Zhang, M. Dan, J. F. Yang, F. X. Wu, L. G. Wang, H. Tang, Z. Q. Liu, *Adv. Funct. Mater*. **2023**, *33*, 2302964.

[4] L. Zhou, J. Feng, B. Qiu, Y. Zhou, J. Lei, M. Xing, L. Wang, Y. Zhou, Y. Liu, J. Zhang, *Appl. Catal. B* **2020**, *267*, 118396

[5] Z. Teng, Q. Zhang, H. Yang, K. Kato, W Yang, Y. R. Lu, S. Liu, C. Wang, A. Yamakata, C. Su, B. Liu, T. Ohno, *Nat. Catal*. **2021**, *4*, 374.

[6] Y. Shiraishi, T. Takii, T. Hagi, S. Mori, Y. Kofuji, Y. Kitagawa, S. Tanaka, S. Ichikawa, T. Hirai, *Nat. Mater*. **2019**, *18*, 985.

[7] Y. Shiraishi, M. Matsumoto, S. Ichikawa, S. Tanaka, T. Hirai, *J. Am. Chem. Soc*. **2021**, *143*, 12590.

[8] Y. Shiraishi, T. Hagi, M. Matsumoto, S. Tanaka, S. Ichikawa, T. Hirai, *Commun. Chem*. **2020**, *3*, 169.

[9] L. Yuan, C. Zhang, J. Wang, C. Liu, C. Yu, *Nano Res*. **2021**, *14*, 3267.

[10] X. Y. Wang, X. W. Yang, C. Zhao, Y. T. Pi, X. B. Li, Z. F. Jia, S. Zhou, J. J. Zhao, L. M. Wu, J. Liu, *Angew. Chem. Int. Ed.* **2023**, *62*, e202302829

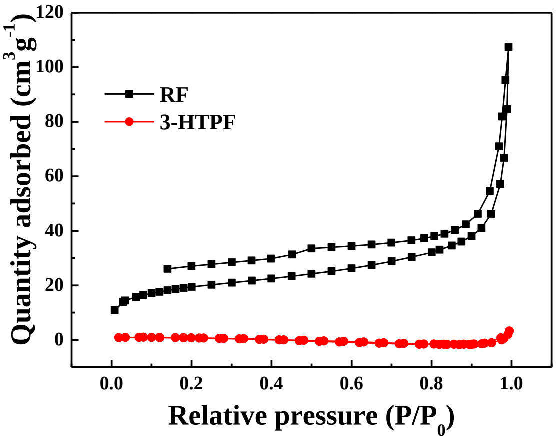

Figure S13. N_2 adsorption and desorption isotherms of 3-HTPF and RF resins.

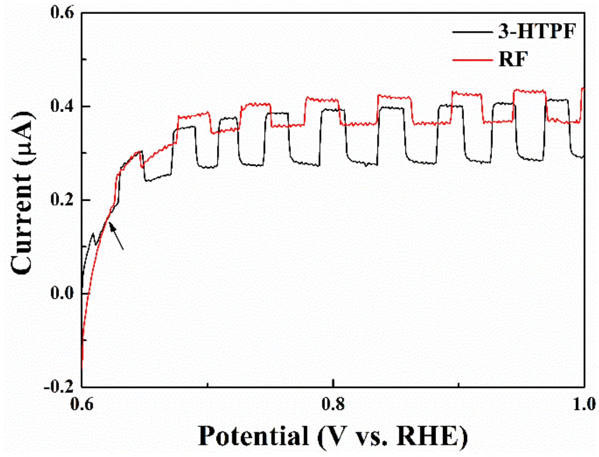

Figure S14. Photocurrent spectra of 3-HTPF and RF resins.

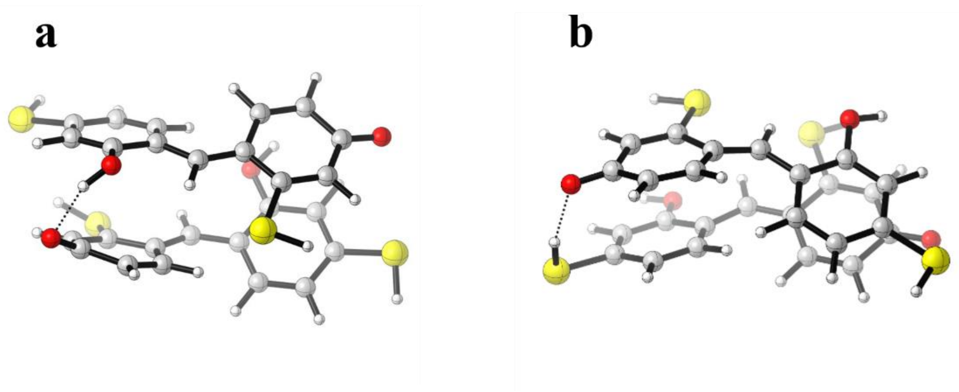

Dipole Moment (Debye): **4.52** **2.12**

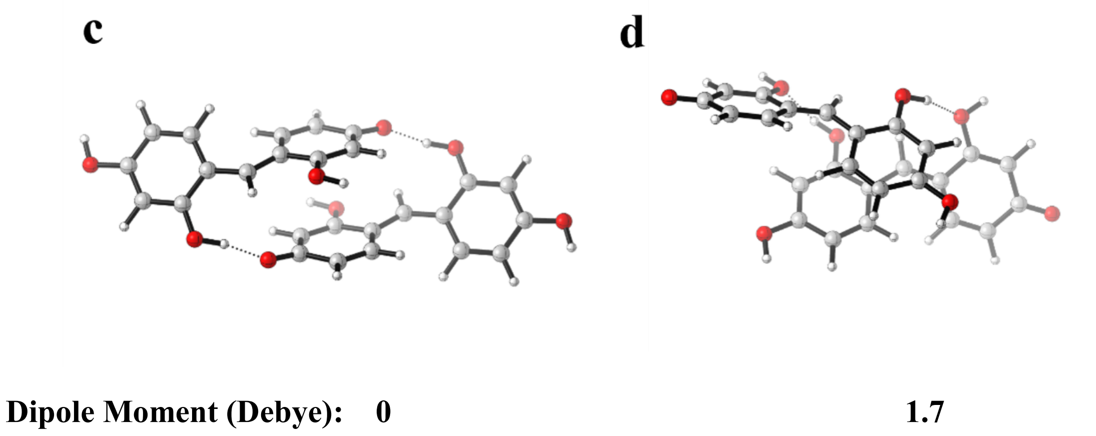

Figure S15. Structural models of 3-HTPF (a-b) and RF (c-d) and corresponding dipole moments.

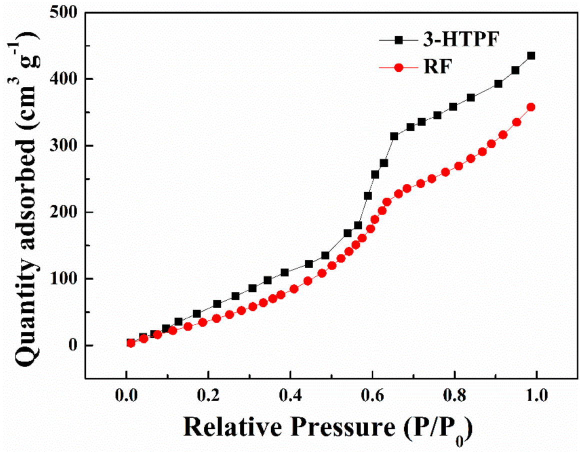

Figure S16. O_2 adsorption isotherms of 3-HTPF and RF at 298 K.

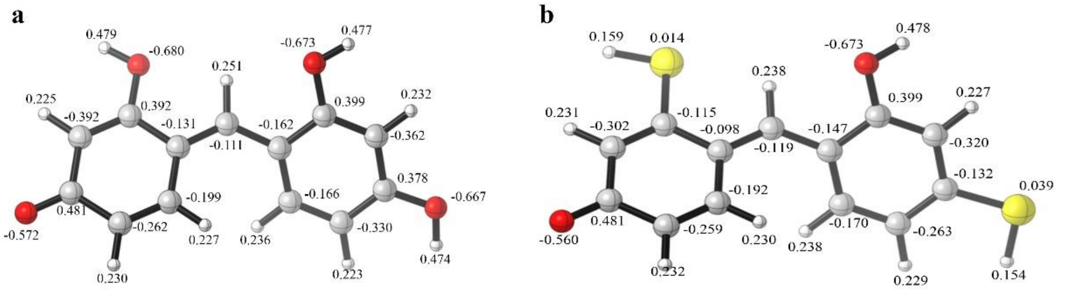

Figure S17. The atomic charges obtained from natural population analysis for RF (a) and 3-HTPF molecular models (b).

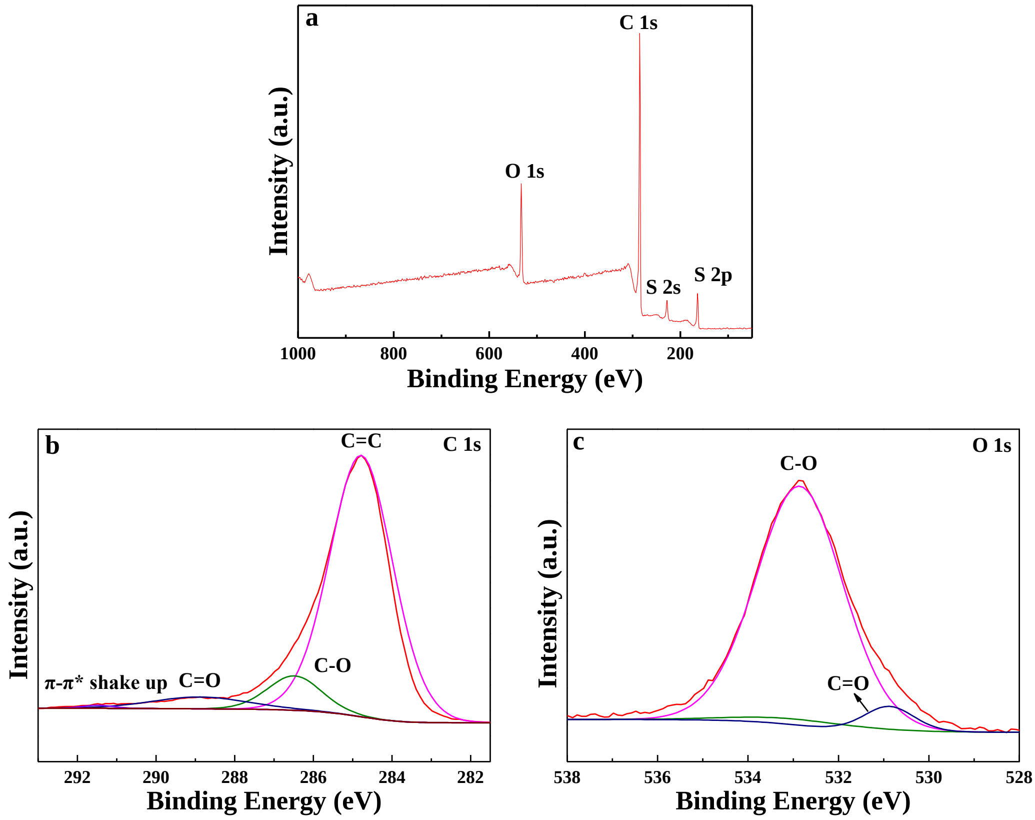

Figure S18. XPS survey spectrum of 3-HTPF after one hour of ORR (a). Corresponding high-resolution C1s (b) and O1s spectra (c).

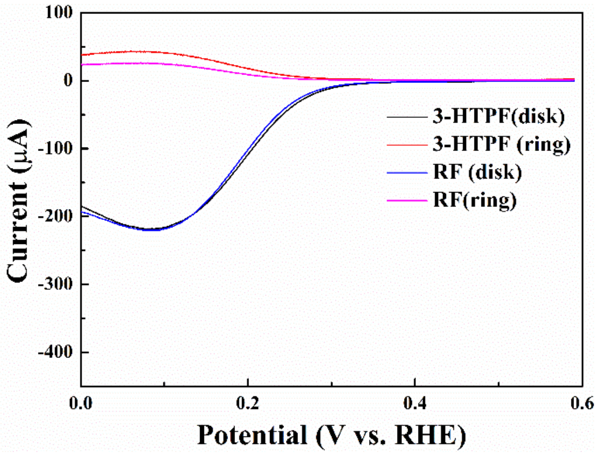

Figure 19. RRDE tests of 3-HTPF and RF resins in O_2-saturated 0.1 M Na_2SO_4 aqueous solution.

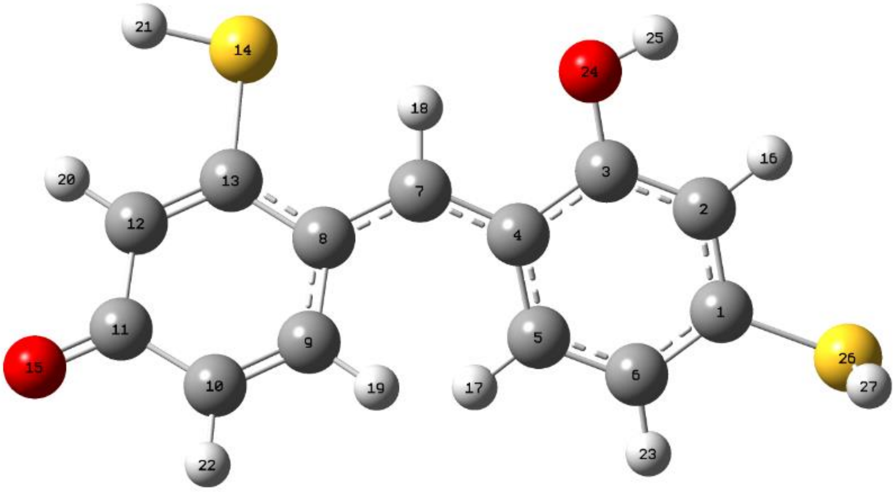

Figure S20. The structural model of 3-HTPF anion.

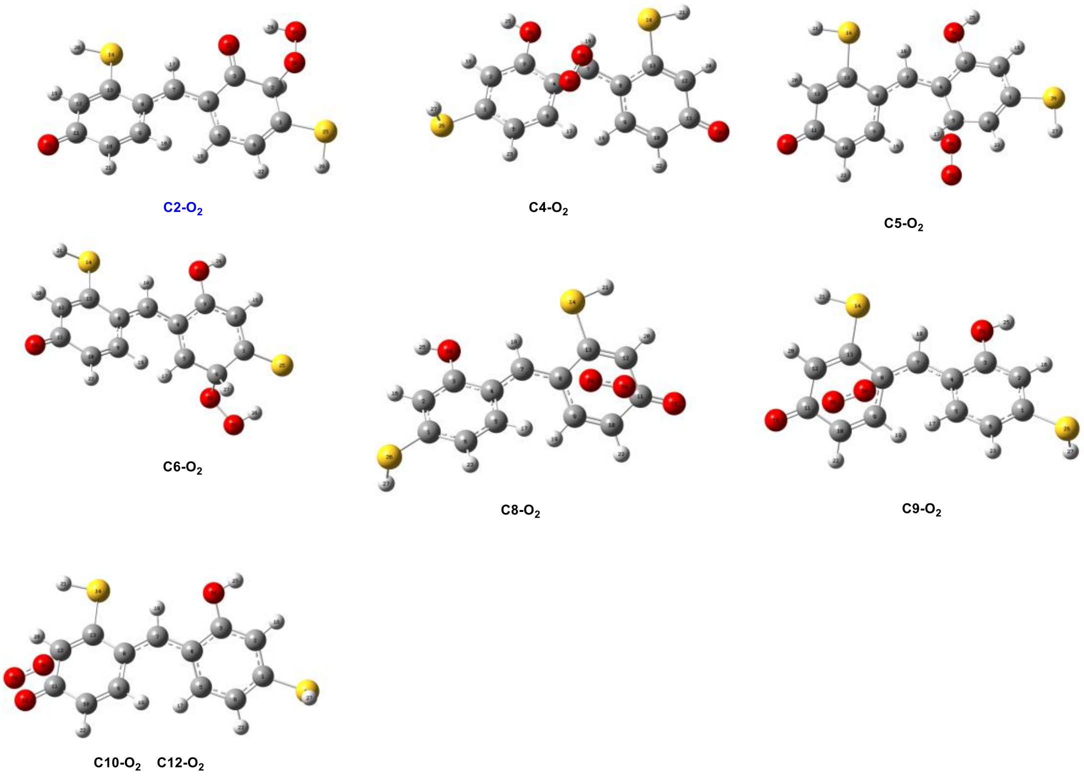

Figure S21. The structures of O_2 molecule adsorbed on 3-HTPF anion at different positions.

The carbon atoms (C1, C3, C11, C13) connected with heteroatoms and carbon atom not within benzene ring structure (C7) are not considered because they are not suitable for ORR reaction. The calculated O_2 adsorption energies versus C2 at different positions of 3-HTPF anion are shown in Table S4.

Table S4. O_2 adsorption energy at different positions of 3-HTPF

Position	Gibbs free energy versus C2 in aqueous solution phase（kcal mol^{-}）
C2	0.0
C4	5.1
C5	13.7
C6	5.3
C8	4.3
C9	9.7
C10 or C12	11.8

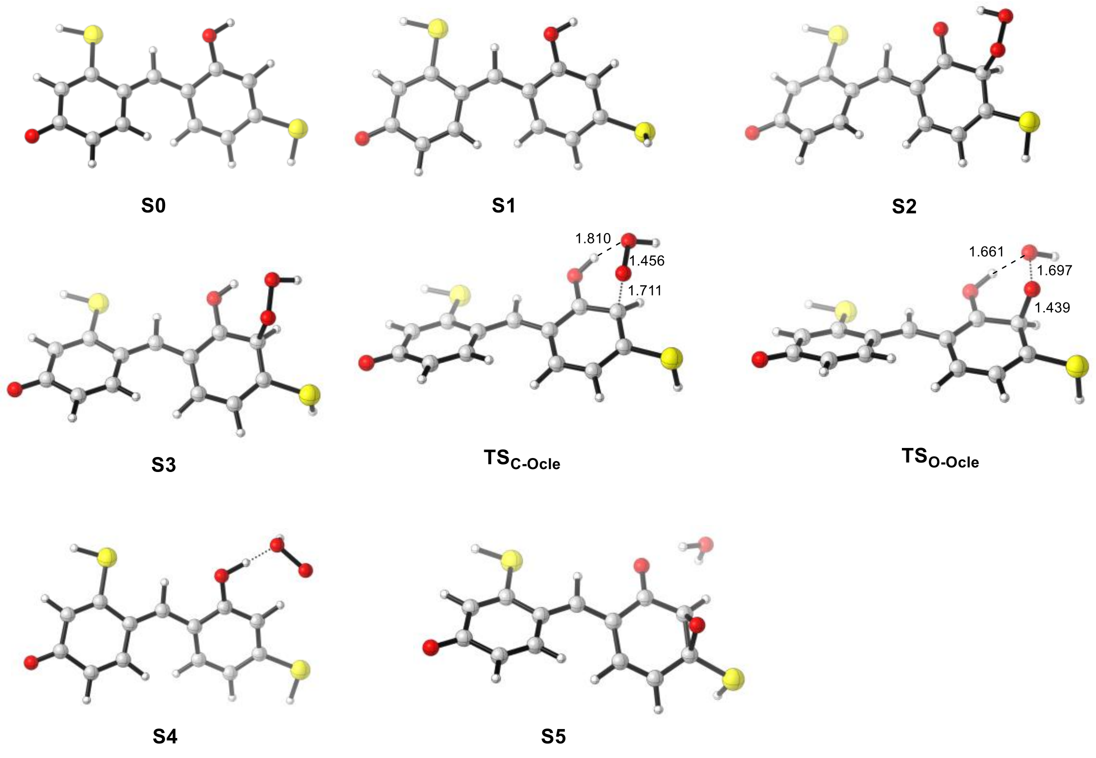

Figure S22. Optimized structures of the ORR pathway of 3-HTPF. TS represents transition state.

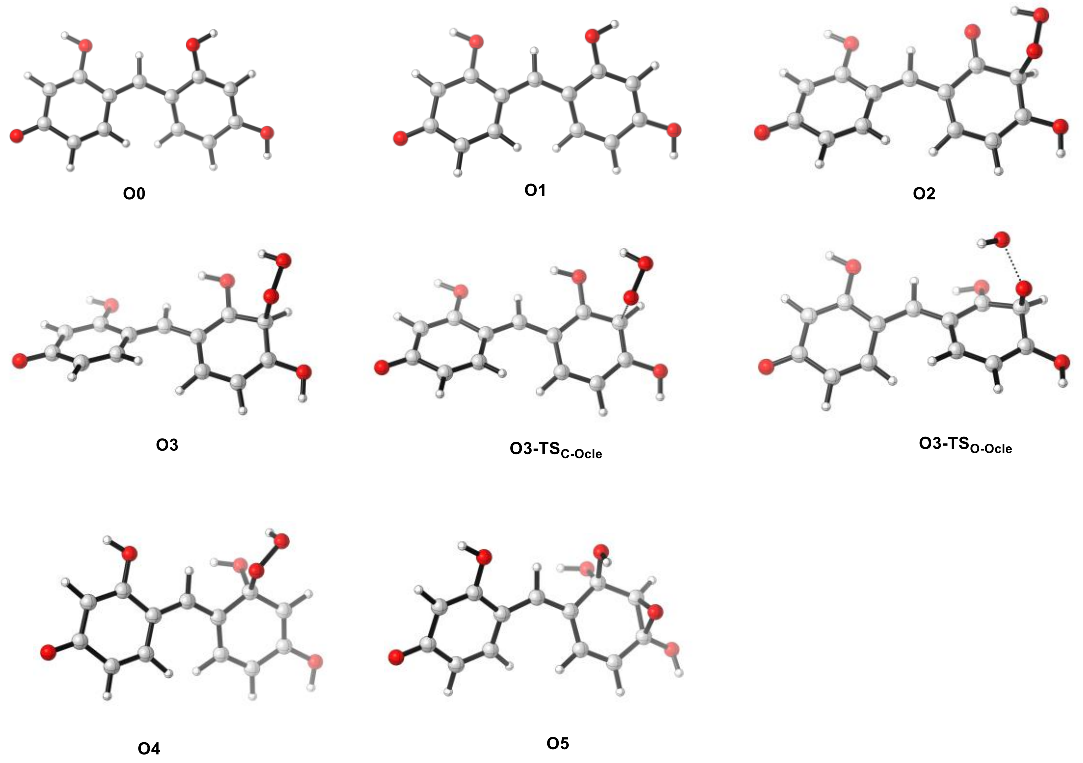

Figure S23. Optimized structures of the ORR pathway of RF.

Energy data (a.u.)

**Structure**	**TCG (**Gibbs free energy correction**)**	Single-point Energy
O_2	-0.016091	-150.327545
**S0**	0.149138	-1448.195690
**S1**	0.145761	-1448.319928
**S2**	0.151254	-1598.663914
**S3**	0.161613	-1599.237297
**S3-TS$_{C-Ocle}$**	0.161702	-1599.237070
**S3-TS$_{O-Ocle}$**	0.160840	-1599.217598
**S4**	0.157570	-1599.280760
**S5**	0.160202	-1599.361292
H_2O	0.003569	-76.439284
OH^-	-0.008548	-75.938316

HOO^-	-0.009174	-151.078802
**O0**	0.163002	-802.299963
**O1**	0.158864	-802.418815
**O2**	0.163983	-952.760509
**O3**	0.173685	-953.326464
**O3-TS$_{C\text{-}Ocle}$**	0.172901	-953.336916
**O3-TS$_{O\text{-}Ocle}$**	0.172027	-953.310180
**O4**	0.175809	-953.382219
**O5**	0.177206	-953.460790

Cartesian coordinates

O_2

O	0.000000000	0.000000000	0.599987000
O	0.000000000	0.000000000	-0.599987000

S0

C	3.740213000	-0.410207000	-0.119797000
C	3.339318000	0.819753000	0.422372000
C	1.991294000	1.179738000	0.442158000
C	0.990869000	0.306760000	-0.064141000
C	1.429494000	-0.910493000	-0.625049000
C	2.770309000	-1.274029000	-0.656606000
C	-0.390857000	0.751539000	-0.051004000
C	-1.539391000	0.006194000	0.037973000
C	-1.537872000	-1.411109000	0.380189000
C	-2.669771000	-2.143214000	0.472807000
C	-3.994640000	-1.547719000	0.218840000
C	-3.996987000	-0.111579000	-0.092122000

C	-2.857658000	0.629490000	-0.164195000
S	-2.883304000	2.363619000	-0.565121000
O	-5.030395000	-2.203251000	0.278974000
H	4.083713000	1.505188000	0.838113000
H	0.696118000	-1.568182000	-1.091960000
H	-0.503498000	1.837934000	-0.104299000
H	-0.578647000	-1.868312000	0.623763000
H	-4.981588000	0.324628000	-0.273760000
H	-4.227404000	2.487704000	-0.513240000
H	-2.656238000	-3.195000000	0.766582000
H	3.065260000	-2.219386000	-1.115374000
O	1.575881000	2.360869000	0.955005000
H	2.336950000	2.887810000	1.232374000
S	5.469938000	-0.785997000	-0.107578000
H	5.369372000	-2.006007000	-0.679409000

S1

C	3.775634000	-0.450539000	-0.128582000
C	3.386899000	0.843966000	0.282538000
C	2.049197000	1.201173000	0.354482000
C	0.992366000	0.279526000	0.032791000
C	1.429530000	-1.005796000	-0.412183000
C	2.771331000	-1.361392000	-0.489526000
C	-0.357752000	0.717429000	0.123028000
C	-1.565619000	-0.027457000	0.105125000
C	-1.657438000	-1.434695000	0.375605000
C	-2.841957000	-2.127596000	0.394145000
C	-4.122743000	-1.489053000	0.129275000
C	-4.026319000	-0.060201000	-0.130799000
C	-2.831563000	0.626350000	-0.130823000

S	-2.794757000	2.389120000	-0.492028000
O	-5.211501000	-2.103049000	0.131047000
H	4.157093000	1.573946000	0.553186000
H	0.679145000	-1.720067000	-0.747378000
H	-0.467623000	1.798478000	0.229564000
H	-0.739079000	-1.962587000	0.636752000
H	-4.971545000	0.447764000	-0.342925000
H	-4.134014000	2.558179000	-0.413579000
H	-2.866437000	-3.194974000	0.633437000
H	3.053900000	-2.354898000	-0.845790000
O	1.676845000	2.458100000	0.750061000
H	2.478354000	2.976859000	0.890705000
S	5.505936000	-0.883188000	-0.239637000
H	5.699203000	-1.328306000	1.033356000

S2

C	3.264982000	-1.050552000	-0.256128000
C	3.069884000	0.429874000	-0.189436000
C	1.618259000	0.894468000	0.049289000
C	0.518988000	-0.047714000	-0.125954000
C	0.852119000	-1.411137000	-0.396330000
C	2.176531000	-1.884560000	-0.414957000
C	-0.777981000	0.504496000	-0.030925000
C	-2.051013000	-0.084593000	0.054853000
C	-2.281712000	-1.465095000	0.397505000
C	-3.526610000	-2.013431000	0.509587000
C	-4.741469000	-1.236565000	0.268337000
C	-4.503370000	0.164717000	-0.066016000
C	-3.246934000	0.710859000	-0.150774000
S	-3.029661000	2.436132000	-0.590852000

O	-5.882383000	-1.719865000	0.348403000
H	0.055827000	-2.117533000	-0.622644000
H	-0.757260000	1.599280000	-0.017478000
H	-1.413040000	-2.077666000	0.638704000
H	-5.398395000	0.762418000	-0.257839000
H	-4.334793000	2.760294000	-0.453195000
H	-3.663600000	-3.056747000	0.806766000
H	2.343695000	-2.953199000	-0.581706000
O	1.472007000	2.084408000	0.371178000
H	3.135357000	2.635619000	0.655612000
S	4.960580000	-1.569863000	-0.247776000
H	4.733424000	-2.800185000	-0.768856000
H	3.397648000	0.894659000	-1.146631000
O	3.921077000	0.949283000	0.833945000
O	4.086347000	2.364693000	0.605350000

S3

C	3.274462000	-0.969097000	-0.346966000
C	3.037829000	0.435278000	-0.070493000
C	1.619696000	0.826989000	-0.158469000
C	0.558719000	-0.092466000	-0.257304000
C	0.877487000	-1.491305000	-0.409682000
C	2.203267000	-1.876744000	-0.455630000
C	-0.759167000	0.467922000	-0.234940000
C	-2.008173000	-0.107267000	-0.029009000
C	-2.202533000	-1.451495000	0.472582000
C	-3.429984000	-1.984180000	0.713259000
C	-4.663855000	-1.235609000	0.459234000
C	-4.464253000	0.126286000	-0.039094000
C	-3.225506000	0.665164000	-0.253498000

S	-3.046626000	2.331627000	-0.882988000
O	-5.790838000	-1.707201000	0.655708000
H	0.091061000	-2.214172000	-0.609828000
H	-0.762707000	1.550099000	-0.392634000
H	-1.307427000	-2.023202000	0.713735000
H	-5.378001000	0.691652000	-0.239124000
H	-4.352340000	2.652531000	-0.744266000
H	-3.544719000	-2.990267000	1.124830000
H	2.450289000	-2.926926000	-0.639928000
O	1.387232000	2.159592000	-0.053071000
H	4.528067000	2.364733000	1.151089000
S	4.961115000	-1.498385000	-0.540728000
H	5.023651000	-1.643157000	-1.900635000
H	3.693689000	1.143218000	-0.622308000
O	3.501891000	0.774560000	1.408008000
O	3.635171000	2.229629000	1.509571000
H	2.151028000	2.530122000	0.436848000

S3-TS$_{C\text{-}Ocle}$

C	3.267725000	-0.980677000	-0.366353000
C	3.029824000	0.409465000	-0.115366000
C	1.630600000	0.816823000	-0.175816000
C	0.560851000	-0.098763000	-0.260692000
C	0.875090000	-1.498654000	-0.404495000
C	2.195739000	-1.895483000	-0.448157000
C	-0.754502000	0.464150000	-0.235009000
C	-2.005000000	-0.106884000	-0.023770000
C	-2.202506000	-1.444404000	0.493747000
C	-3.431221000	-1.973464000	0.736078000
C	-4.663589000	-1.227058000	0.468134000

C	-4.460775000	0.128944000	-0.044295000
C	-3.220612000	0.664294000	-0.260178000
S	-3.037622000	2.322924000	-0.908505000
O	-5.791477000	-1.695989000	0.665483000
H	0.082872000	-2.220635000	-0.585560000
H	-0.755053000	1.545665000	-0.398000000
H	-1.309878000	-2.014370000	0.747770000
H	-5.373106000	0.692912000	-0.254349000
H	-4.342165000	2.649290000	-0.771587000
H	-3.548229000	-2.973786000	1.160793000
H	2.437362000	-2.949913000	-0.612279000
O	1.403451000	2.144792000	-0.057987000
H	4.516540000	2.404779000	1.125945000
S	4.958162000	-1.518422000	-0.539167000
H	4.986269000	-1.776272000	-1.880316000
H	3.719903000	1.125899000	-0.597530000
O	3.482567000	0.837459000	1.478668000
O	3.624225000	2.286765000	1.490952000
H	2.176972000	2.510441000	0.427982000

S3-TS$_{O\text{-}Ocle}$

C	3.304921000	-0.974396000	-0.305899000
C	3.058973000	0.461753000	-0.121912000
C	1.607778000	0.846941000	-0.153528000
C	0.557462000	-0.078221000	-0.242713000
C	0.902958000	-1.481214000	-0.374770000
C	2.223303000	-1.876580000	-0.381480000
C	-0.763313000	0.470979000	-0.208032000
C	-2.013205000	-0.113002000	-0.024807000
C	-2.212203000	-1.467749000	0.444237000

C	-3.441618000	-2.005023000	0.667191000
C	-4.673751000	-1.251153000	0.423121000
C	-4.470055000	0.120080000	-0.045761000
C	-3.229555000	0.662787000	-0.241853000
S	-3.047527000	2.341694000	-0.836987000
O	-5.802112000	-1.727084000	0.602262000
H	0.121345000	-2.215829000	-0.548721000
H	-0.769628000	1.556312000	-0.341339000
H	-1.322121000	-2.048655000	0.680443000
H	-5.382031000	0.690187000	-0.240311000
H	-4.352974000	2.661666000	-0.694337000
H	-3.558297000	-3.020545000	1.054286000
H	2.462354000	-2.936597000	-0.510232000
O	1.398772000	2.157014000	-0.007491000
H	4.397490000	2.531342000	1.107049000
S	4.985508000	-1.508002000	-0.492423000
H	5.036622000	-1.671711000	-1.852317000
H	3.636111000	1.133442000	-0.801930000
O	3.557183000	0.671717000	1.211666000
O	3.522745000	2.346796000	1.484136000
H	2.200565000	2.502565000	0.490605000

S4

C	3.079476000	-1.382540000	-0.233712000
C	2.964319000	-0.032934000	0.069650000
C	1.677414000	0.552928000	0.126625000
C	0.498696000	-0.242075000	-0.116198000
C	0.683854000	-1.612305000	-0.447568000
C	1.938514000	-2.182010000	-0.511175000
C	-0.764197000	0.424306000	-0.085887000

C	-2.054983000	-0.069475000	0.060322000
C	-2.349283000	-1.407348000	0.529540000
C	-3.614006000	-1.879398000	0.683037000
C	-4.788876000	-1.059485000	0.366386000
C	-4.488161000	0.301905000	-0.079635000
C	-3.212255000	0.777815000	-0.207548000
S	-2.898209000	2.441157000	-0.781043000
O	-5.946623000	-1.476822000	0.481427000
H	-0.183212000	-2.214517000	-0.719235000
H	-0.665265000	1.510763000	-0.180374000
H	-1.505709000	-2.029985000	0.827959000
H	-5.356805000	0.920854000	-0.317268000
H	-4.185836000	2.843122000	-0.695111000
H	-3.808314000	-2.879699000	1.077975000
H	2.051358000	-3.229345000	-0.801362000
O	1.528591000	1.825500000	0.417610000
H	3.921344000	3.147307000	-0.501899000
S	4.720136000	-2.070662000	-0.295282000
H	4.342213000	-3.341734000	-0.564944000
H	3.842496000	0.630578000	0.330792000
O	4.776571000	2.053753000	0.843222000
O	3.703685000	3.003434000	0.429582000
H	2.476027000	2.331503000	0.466946000

S5

C	3.261539000	-1.108784000	-0.004806000
C	3.038916000	0.315088000	0.372642000
C	1.651132000	0.895290000	0.295054000
C	0.550895000	-0.023127000	0.039260000
C	0.860234000	-1.369023000	-0.402026000

C	2.108061000	-1.894458000	-0.485837000
C	-0.744033000	0.523530000	0.094695000
C	-2.017251000	-0.070905000	0.093517000
C	-2.253007000	-1.464271000	0.377275000
C	-3.496151000	-2.025289000	0.396998000
C	-4.702423000	-1.247998000	0.110544000
C	-4.459003000	0.167605000	-0.155631000
C	-3.205340000	0.725579000	-0.145167000
S	-2.977313000	2.467562000	-0.504972000
O	-5.840317000	-1.741996000	0.106227000
H	0.026719000	-1.975326000	-0.760065000
H	-0.724633000	1.617506000	0.162552000
H	-1.393763000	-2.076826000	0.652960000
H	-5.346978000	0.765563000	-0.376684000
H	-4.287504000	2.783732000	-0.400187000
H	-3.641702000	-3.077506000	0.655773000
H	2.288476000	-2.887130000	-0.900303000
O	1.524730000	2.125863000	0.431865000
H	3.876012000	3.375960000	-0.807415000
S	4.923346000	-1.631255000	-0.555117000
H	4.644037000	-1.552113000	-1.877293000
H	3.843812000	1.053663000	0.285424000
O	3.259916000	-0.710373000	1.355539000
O	3.927087000	3.446636000	0.155351000
H	3.046248000	3.091108000	0.415680000

H_2O

O	0.000000000	0.000000000	0.120211000
H	0.000000000	0.757010000	-0.480842000
H	0.000000000	-0.757010000	-0.480842000

OH^-

O	0.000000000	0.000000000	0.109834000
H	0.000000000	0.000000000	-0.878670000

HOO^-

H	-0.900253000	-0.838146000	0.000000000
O	0.056266000	0.809863000	0.000000000
O	0.056266000	-0.705095000	0.000000000

O0

C	-3.880501000	-0.748454000	0.084384000
C	-3.626474000	0.566464000	-0.324062000
C	-2.326448000	1.069806000	-0.295030000
C	-1.231380000	0.264216000	0.125362000
C	-1.531081000	-1.045814000	0.551969000
C	-2.824135000	-1.555906000	0.535202000
C	0.092804000	0.856991000	0.166513000
C	1.314968000	0.252064000	0.026892000
C	1.516785000	-1.127874000	-0.392870000
C	2.744867000	-1.684041000	-0.492706000
C	3.974554000	-0.924457000	-0.177876000
C	3.770497000	0.477462000	0.193281000
C	2.531471000	1.035268000	0.274426000
O	2.326145000	2.329245000	0.613110000
O	5.088100000	-1.437196000	-0.246797000
O	-5.160595000	-1.179131000	0.035882000
O	-2.048172000	2.336790000	-0.679940000
H	-4.461448000	1.182693000	-0.665797000
H	-0.728474000	-1.663921000	0.953696000

H	0.107725000	1.937215000	0.322356000
H	0.637005000	-1.704188000	-0.679642000
H	4.670444000	1.061343000	0.406646000
H	3.181847000	2.757062000	0.752877000
H	-5.205591000	-2.096758000	0.336111000
H	-2.866581000	2.795587000	-0.910899000
H	-3.022619000	-2.570531000	0.890741000
H	2.888297000	-2.710510000	-0.837043000

O1

C	-3.936422000	-0.775075000	0.092188000
C	-3.691943000	0.582210000	-0.182523000
C	-2.393025000	1.076049000	-0.207187000
C	-1.243875000	0.245033000	0.028456000
C	-1.548570000	-1.114607000	0.327085000
C	-2.853404000	-1.614357000	0.355229000
C	0.057823000	0.830804000	-0.002690000
C	1.338664000	0.218239000	-0.033678000
C	1.623538000	-1.153731000	-0.338907000
C	2.895722000	-1.679342000	-0.370546000
C	4.082684000	-0.883133000	-0.093105000
C	3.787263000	0.512312000	0.186356000
C	2.508178000	1.024803000	0.206275000
O	2.294200000	2.357923000	0.473377000
O	5.245026000	-1.348459000	-0.105626000
O	-5.243637000	-1.208125000	0.093952000
O	-2.162700000	2.398919000	-0.471198000
H	-4.541702000	1.242394000	-0.384063000
H	-0.731841000	-1.783480000	0.590867000
H	0.068037000	1.919670000	0.014124000

H	0.789221000	-1.801899000	-0.610669000
H	4.645398000	1.164581000	0.392666000
H	3.165084000	2.759015000	0.585364000
H	-5.234548000	-2.152394000	0.293253000
H	-3.017575000	2.833403000	-0.578149000
H	-3.027796000	-2.668777000	0.599335000
H	3.062806000	-2.729823000	-0.628490000

O2

C	3.300239000	-1.355000000	-0.257235000
C	3.269191000	0.132321000	-0.230250000
C	1.858197000	0.754658000	-0.176827000
C	0.672792000	-0.092264000	-0.218823000
C	0.863224000	-1.506082000	-0.322788000
C	2.143566000	-2.095987000	-0.334179000
C	-0.563961000	0.592738000	-0.185022000
C	-1.888828000	0.153479000	-0.037163000
C	-2.307461000	-1.165918000	0.363469000
C	-3.617049000	-1.526934000	0.502922000
C	-4.719196000	-0.595476000	0.245367000
C	-4.288812000	0.743720000	-0.131348000
C	-2.967175000	1.095781000	-0.253235000
O	-2.605591000	2.364417000	-0.606777000
O	-5.913551000	-0.919611000	0.352401000
O	4.557733000	-1.889754000	-0.307807000
O	1.830354000	1.993728000	-0.055172000
H	0.001299000	-2.154213000	-0.461983000
H	-0.449334000	1.672722000	-0.299395000
H	-1.533774000	-1.889737000	0.617418000
H	-5.079717000	1.477976000	-0.322746000

H	-3.415661000	2.883277000	-0.689920000
H	4.468747000	-2.851734000	-0.329446000
H	3.457299000	2.342443000	0.524103000
H	2.223576000	-3.184764000	-0.440126000
H	-3.897920000	-2.530354000	0.834642000
H	3.789943000	0.537896000	-1.124445000
O	4.007615000	0.589492000	0.915127000
O	4.361839000	1.972512000	0.690048000

O3

C	3.254097000	-1.217060000	-0.531447000
C	3.231887000	0.190413000	-0.137555000
C	1.884523000	0.729374000	-0.310871000
C	0.706787000	-0.051024000	-0.342244000
C	0.825541000	-1.481687000	-0.449824000
C	2.108664000	-2.008501000	-0.588726000
C	-0.556787000	0.608370000	-0.424711000
C	-1.842041000	0.174598000	-0.113443000
C	-2.151369000	-1.038377000	0.609919000
C	-3.426149000	-1.427703000	0.883032000
C	-4.594014000	-0.639161000	0.465386000
C	-4.270559000	0.605123000	-0.226789000
C	-2.983392000	0.990450000	-0.486941000
O	-5.757377000	-0.990728000	0.701685000
H	-0.051765000	-2.095083000	-0.638126000
H	-0.535972000	1.633332000	-0.814500000
H	-1.306120000	-1.626654000	0.968976000
H	-5.116576000	1.228306000	-0.536405000
H	-3.635620000	-2.334877000	1.455694000
H	2.231476000	-3.076105000	-0.817088000

O	1.859046000	2.119096000	-0.171736000
H	3.392815000	2.187697000	1.318430000
H	4.017189000	0.799489000	-0.626850000
O	3.665692000	0.320338000	1.342556000
O	4.184421000	1.662747000	1.536269000
H	0.941984000	2.403054000	-0.074557000
O	4.512628000	-1.732822000	-0.770397000
H	4.398365000	-2.649473000	-1.050924000
O	-2.703921000	2.161049000	-1.131673000
H	-3.542830000	2.603996000	-1.313000000

O3-TS$_{C\text{-}Ocle}$

C	3.253017000	-1.280996000	-0.534016000
C	3.189398000	0.118446000	-0.304955000
C	1.885408000	0.676099000	-0.367238000
C	0.693515000	-0.108161000	-0.353918000
C	0.830726000	-1.533456000	-0.404757000
C	2.102979000	-2.081399000	-0.517202000
C	-0.560286000	0.567282000	-0.420178000
C	-1.853490000	0.154454000	-0.114422000
C	-2.192220000	-1.060264000	0.592648000
C	-3.474957000	-1.417247000	0.870295000
C	-4.623527000	-0.592638000	0.467052000
C	-4.270846000	0.650386000	-0.212846000
C	-2.975381000	1.004029000	-0.474781000
O	-5.793757000	-0.918581000	0.702149000
H	-0.048383000	-2.164450000	-0.509897000
H	-0.523219000	1.592279000	-0.805795000
H	-1.365952000	-1.677476000	0.945447000
H	-5.101412000	1.298802000	-0.511692000

H	-3.706024000	-2.326650000	1.430729000
H	2.214869000	-3.164930000	-0.650485000
O	1.851845000	2.048683000	-0.300163000
H	3.587570000	2.379160000	1.174119000
H	4.034345000	0.748821000	-0.598816000
O	3.650086000	0.533262000	1.520330000
O	4.328449000	1.802138000	1.423892000
H	0.955920000	2.325785000	-0.072113000
O	-2.666380000	2.173744000	-1.105663000
H	-3.492076000	2.645543000	-1.274622000
O	4.506847000	-1.821819000	-0.700318000
H	4.401629000	-2.775514000	-0.802787000

O3-TS$_{O\text{-}Ocle}$

C	-3.415533000	-1.208774000	0.198536000
C	-3.326906000	0.273225000	0.236281000
C	-1.933798000	0.708507000	0.574574000
C	-0.816249000	-0.018044000	0.084409000
C	-1.067568000	-1.345673000	-0.434914000
C	-2.317309000	-1.929818000	-0.306369000
C	0.453003000	0.613840000	0.171044000
C	1.756971000	0.124471000	0.071737000
C	2.127175000	-1.271299000	0.064072000
C	3.419987000	-1.693378000	-0.008482000
C	4.547913000	-0.756579000	-0.094562000
C	4.166946000	0.651369000	-0.062915000
C	2.863160000	1.062273000	0.022806000
O	5.725614000	-1.132938000	-0.172058000
H	-0.281856000	-1.859323000	-0.985834000
H	0.409784000	1.700045000	0.310967000

H	1.324832000	-2.001508000	0.164688000
H	4.981945000	1.381698000	-0.111860000
H	3.674595000	-2.756231000	0.012542000
H	-2.464033000	-2.971148000	-0.623623000
O	-1.818156000	1.678710000	1.490343000
H	-2.348629000	1.918748000	-1.913681000
H	-4.046017000	0.746142000	0.937520000
O	-3.614017000	0.423847000	-1.087675000
O	-2.969444000	2.264868000	-1.255175000
H	-0.888038000	1.750770000	1.750698000
O	2.533414000	2.385897000	0.061859000
H	3.352746000	2.895812000	0.024811000
O	-4.588132000	-1.793269000	0.601711000
H	-4.616321000	-2.682652000	0.224436000

O4

C	3.482354000	-1.411959000	-0.084638000
C	3.430637000	-0.068087000	0.060325000
C	2.145729000	0.680285000	-0.064429000
C	0.898714000	-0.181713000	-0.099274000
C	1.066108000	-1.585738000	-0.307660000
C	2.291427000	-2.193987000	-0.324251000
C	-0.321545000	0.493271000	-0.029811000
C	-1.666274000	0.050662000	0.039547000
C	-2.107812000	-1.269017000	0.391541000
C	-3.426848000	-1.630670000	0.443738000
C	-4.510227000	-0.694685000	0.136253000
C	-4.051792000	0.648640000	-0.185804000
C	-2.721173000	0.995808000	-0.219809000
O	-5.711629000	-1.016152000	0.164998000

H	0.177350000	-2.184444000	-0.504149000
H	-0.234433000	1.583515000	-0.023763000
H	-1.353047000	-1.998562000	0.687646000
H	-4.825176000	1.391205000	-0.414710000
H	-3.726588000	-2.637315000	0.748525000
H	2.384499000	-3.265025000	-0.532443000
O	2.232694000	1.533571000	-1.236086000
H	2.909521000	2.827462000	0.028967000
H	4.327860000	0.515587000	0.262042000
O	1.960986000	1.585379000	1.049502000
O	2.989460000	2.600591000	0.979400000
H	1.317938000	1.663595000	-1.530331000
O	-2.336502000	2.274395000	-0.534346000
H	-3.142140000	2.797644000	-0.631270000
O	4.689759000	-2.062781000	-0.001048000
H	4.510286000	-3.010552000	-0.004236000

O5

C	-3.570371000	-0.966804000	-0.201471000
C	-3.394571000	0.440142000	0.136418000
C	-2.014600000	1.026681000	0.390336000
C	-0.854315000	0.080624000	0.065263000
C	-1.138342000	-1.304448000	-0.203720000
C	-2.384000000	-1.835732000	-0.320486000
C	0.409810000	0.657350000	0.069124000
C	1.728867000	0.119403000	0.058769000
C	2.094013000	-1.227901000	0.383577000
C	3.386759000	-1.683223000	0.371659000
C	4.517787000	-0.825411000	0.016565000
C	4.137265000	0.546520000	-0.280752000

C	2.832003000	0.986306000	-0.251226000
O	5.695510000	-1.229955000	-0.010059000
H	-0.286086000	-1.956323000	-0.396276000
H	0.389318000	1.752072000	0.063508000
H	1.306085000	-1.908310000	0.711034000
H	4.948042000	1.237116000	-0.542193000
H	3.626899000	-2.711639000	0.656654000
H	-2.532290000	-2.888536000	-0.579199000
O	-2.018376000	1.375540000	1.753577000
H	-1.844050000	1.987695000	-1.269337000
H	-4.195144000	0.973018000	0.666780000
O	-3.685617000	0.077979000	-1.214813000
O	-1.917193000	2.248816000	-0.340178000
H	-1.090386000	1.409915000	2.022087000
O	2.527659000	2.293988000	-0.537273000
H	3.364453000	2.752004000	-0.685915000
O	-4.768116000	-1.563055000	0.142927000
H	-4.859624000	-2.354569000	-0.404931000
